# Population dynamics of Baltic herring since the Viking Age revealed by ancient DNA and genomics

**DOI:** 10.1073/pnas.2208703119

**Published:** 2022-10-25

**Authors:** Lane M. Atmore, Lourdes Martínez-García, Daniel Makowiecki, Carl André, Lembi Lõugas, James H. Barrett, Bastiaan Star

**Affiliations:** ^a^Centre for Ecological and Evolutionary Synthesis, Department of Biosciences, University of Oslo, 0316 Oslo, Norway;; ^b^Department of Environmental Archaeology and Human Paleoecology, Institute of Archaeology, Nicolaus Copernicus University, 87-100 Toruń, Poland;; ^c^Department of Marine Sciences–Tjärnö, University of Gothenburg, 452 96 Strömstad, Sweden;; ^d^Archaeological Research Collection, Tallinn University, 10120 Tallinn, Estonia;; ^e^Department of Archaeology and Cultural History, NTNU University Museum, Norwegian University of Science and Technology (NTNU), 7012 Trondheim, Norway

**Keywords:** ecology, history, ancient DNA, fisheries, sustainability

## Abstract

The world’s oceans are currently facing major stressors in the form of overexploitation and anthropogenic climate change. The Baltic Sea was home to the first “industrial” fishery ∼800 y ago targeting the Baltic herring, a species that is still economically and culturally important today. Yet, the early origins of marine industries and the long-term ecological consequences of historical and contemporary fisheries remain debated. Here, we study long-term population dynamics of Baltic herring to evaluate the past impacts of humans on the marine environment. We combine modern whole-genome data with ancient DNA (aDNA) to identify the earliest-known long-distance herring trade in the region, illustrating that extensive fish trade began during the Viking Age. We further resolve population structure within the Baltic and observe demographic independence for four local herring stocks over at least 200 generations. It has been suggested that overfishing at Øresund in the 16th century resulted in a demographic shift from autumn-spawning to spring-spawning herring dominance in the Baltic. We show that while the Øresund fishery had a negative impact on the western Baltic herring stock, the demographic shift to spring-spawning dominance did not occur until the 20th century. Instead, demographic reconstructions reveal population trajectories consistent with expected impacts of environmental change and historical reports on shifting fishing targets over time. This study illustrates the joint impact of climate change and human exploitation on marine species as well as the role historical ecology can play in conservation and management policies.

## Significance

A growing body of research indicates that ocean ecologies have been more impacted by human exploitation and for longer than previously understood. Here, we evaluate human impact on Baltic herring, an ecologically, culturally, and economically important species with an iconic history of exploitation. We observe genomic evidence of the earliest long-distance trade for this species, providing evidence for a longer exploitation history than previously understood. Observations of serial exploitation are consistent with classic patterns of resource depletion. Our results illustrate the importance of including knowledge regarding long-term population dynamics, including differential stock responses to climate change, in sustainable management strategies, as efforts to achieve food security by aquaculture- and marine-based industries are demanding ever-increasing resources from the oceans.

Atlantic herring, *Clupea harengus*, has made a dramatic mark on European history; the fate of nations and peoples has depended on the Atlantic herring trade (1–4). By the medieval era, herring was traded in urban areas across Europe, becoming one of the first true commodity goods and arguably marking the advent of the modern market economy (5). Today, Atlantic herring is one of the most important fisheries for several countries across the Atlantic and into the Baltic (6, 7). We here assess the demographic trajectories of four key Baltic herring stocks, relating these trajectories to historical data on changing fishing pressures and climate proxies. The Baltic Sea has long been under intense exploitation pressure (8) and is particularly vulnerable to both climate change (9) and intensified extraction (10). It therefore provides an excellent opportunity to evaluate shifting impacts of human exploitation and changing climate on marine species (11). We further explore evidence for earlier long-distance trade and exploitation in the Baltic than previously considered. This long, interconnected history between herring, humans, and the climate must be jointly considered when determining crucial stock assessment and fishing measures, such as maximum sustainable yield (MSY) and total allowable catch.

## Historical Exploitation

During the Middle Ages, diets throughout Europe shifted to rely more heavily on marine fish (12). Increased demand for fish protein followed the rise of urbanism and the spread of Christian fasting practices in Europe (5, 12, 13). Herring was a particularly valued species at the time due to its ability to be preserved and sold in quantity at market, its massive spawning aggregations that provided easy capture, and its high fat content (14, 15). Long-distance herring trade, however, was limited by economical access to salt, which is required to keep fresh-caught herring from spoiling (16). One of the earliest fisheries that had access to both salt and coastal herring spawning aggregations occurred in the western Baltic. According to historical data, the earliest commercial herring fishing operations in the Baltic took place on the island of Rügen in the early 12th century (1), an operation that was succeeded by the first fishery in Europe to reach the level of a true industry: the Øresund fishery (8, 17, 18).

**Fig. 1.**
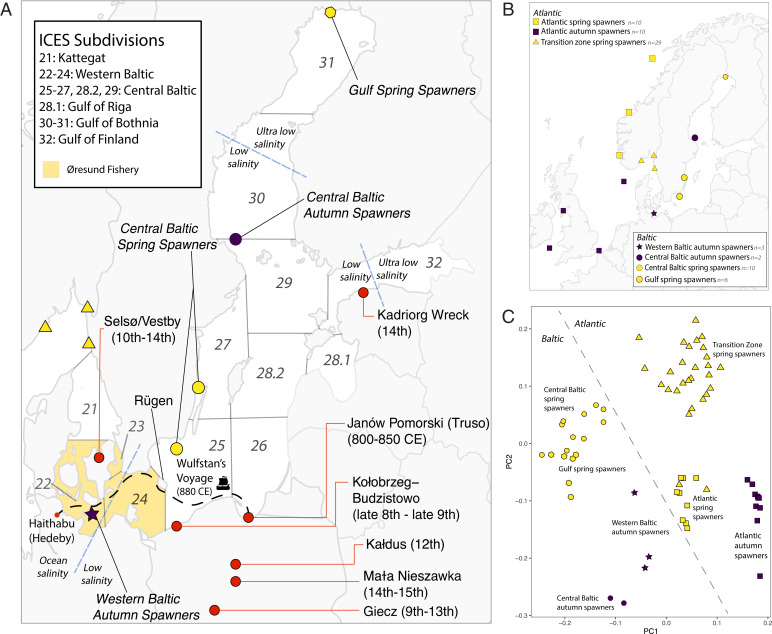
Archaeological sites, sample locations, and population structure of Baltic herring. (*A*) Baltic Sea with ICES management subdivisions indicated. Named regions corresponding to ICES subdivisions are noted (*Inset*). Spring- and autumn-spawning herring occur in all subdivisions. Archaeological sites and their dates are noted as well as the sampling locations for modern genome data. The approximate area targeted by the Øresund fishery (broadly defined) in the Middle Ages is highlighted in yellow (ICES subdivisions 22 to 24), although it is concentrated in subdivisions 23 and 24. Approximate locations of salinity boundaries between high (ocean), low, and ultra-low salinity are marked by blue dashed lines. Wulfstan’s voyage from Hedeby to Truso c. 880 CE is noted in black. (*B*) Sample locations of herring specimens used for this study. The final dataset contained genome sequences generated for this study *(n* = 53) and publicly available genome sequences (*n* = 22). Color denotes spawning season, indicating near-complete coverage of the major herring populations identified by Han et al. (37). (*C*) PCA of the entire modern nuclear genome using 10,368,446 SNPs. The modern genome shows segregation on PC1 based on Atlantic vs. Baltic, and spawning season on PC1 and PC2. The Atlantic spring spawners are divided into two groups, with the transition zone between the Baltic and Atlantic separating on PC2. This is likely due to the presence of Norwegian fjord herring in the transition-zone cluster, which show signatures of adaptation similar to the Baltic herring due to the brackish fjord conditions (87). Winter- and autumn-spawning specimens from the Atlantic cluster together. GSS and CBSS show substructure within the Baltic spring-spawning metapopulation and WBAS cluster away from CBAS.

The Øresund fishery (here broadly defined) operated between Denmark and southern Sweden, corresponding to International Council for the Exploration of the Sea (ICES) subdivisions 22 to 24, with most activity concentrated in subdivisions 23 and 24 (7, 16) (Fig. 1*A*). During the peak of the Øresund fishery, herring were traded across Europe, reaching as far away as Italy and northern Norway (19). Yet, the nature, origin, and timing of onset of this commercial fishery remain unclear (4, 8). The famous account of the Anglo-Saxon Wulfstan (20) reports a voyage c. 880 CE between the trading ports Hedeby (in modern Schleswig-Holstein, Germany) and Truso (identified at the archaeological site of Janów Pomorski) near Elbląg in Poland. Zooarchaeological evidence from a herring bone assemblage dated c. 800 to 850 CE from Janów Pomorski might be indicative of such early medieval fish trade in the same direction due to the absence of cleithra—a bone element often removed during medieval commercial processing (4, 21)—yet the origin of these bones remains unknown. Furthermore, some have hypothesized that the Øresund fishery targeted an Atlantic herring population that had made its way into the transition zone between the Baltic and the Atlantic (13). These hypotheses can now be tested using ancient DNA (aDNA).

Under the control of Denmark and the Hanseatic League, the Øresund fishery was most active during the 13th to 16th centuries. Exact quantities of catch are still under debate (8, 16), but the Øresund fishery likely surpassed contemporary western Baltic fisheries, with catches estimated at up to 50,000 t per annum at its peak (8, 16, 17). In contrast, early–20th century landings from the Øresund were between 100 and 10,000 t (18), and more recent landings have been on the order of 10,000 to 20,000 t (7). Unlike current Baltic fisheries, the Øresund fishery focused exclusively on autumn-spawning herring, with a restricted fishing season between August and October to target the spawning aggregation (16, 22). Today, there are autumn spawners extant in the Baltic, but none of these populations are large enough to support a commercial industry (7). Instead, contemporary Baltic herring fisheries target the smaller-bodied spring spawners, which are ecologically distinct from autumn spawners (23). Although both autumn- and spring-spawning populations coexist wherever herring are found, in each area only one of these distinct ecotypes appears to dominate numerically (e.g., spring spawners in the Norwegian Sea and autumn spawners in the North Sea) (24, 25). This alternating pattern of dominance in today’s oceans, and the known historical shift toward targeting spring- instead of autumn-spawning stocks, indicates that a dramatic demographic and ecological shift has occurred in the Baltic ecosystem. Nonetheless, the timing and nature of this shift are currently poorly characterized.

The Øresund fishery collapsed in the late 16th century, with only marginal operations continuing into the 17th century (16). After this, commercial herring fishing operations were limited in the area until the 20th century (16, 26). European herring production moved to the North Sea (27), a transition that is generally linked to the decline in the Øresund fishery landings. Yet, whether this decline was due to ecological changes or driven by market and political factors is still under debate (28). A recent study argued that the impetus for the collapse of the Øresund fishery was the disappearance of the western Baltic autumn-spawning herring due to overfishing as the stock was pushed past modern MSY estimates (5, 7, 16). This study further proposed that such collapse may have led to a demographic dominance of spring-spawning herring as early as the 16th century (16). Yet, catch records indicate that autumn spawners constituted up to 90% of Baltic herring landings as late as 1927 (29), and autumn-spawning fisheries were supported in the gulfs of Finland, Riga, and Bothnia into the 1970s (30). Past research regarding herring stock collapses has shown that the species is vulnerable to overfishing despite its natural abundance (26, 30); the Baltic autumn spawners’ collapse in the early 20th century has been definitively linked to fishing pressure rather than contemporaneous factors such as eutrophication (30). Thus, the medieval Øresund fishery could well have had a real ecological impact on the herring stock. However, whether this fishery caused a Baltic-wide demographic shift, a short-term local extirpation, or more lasting impacts remains unclear. The impacts of fisheries that succeeded the medieval Øresund industry are also uncharted. Finally, changing climate in the Baltic during periods such as the Medieval Climate Anomaly (MCA) and the Little Ice Age (LIA) likely contributed to herring population dynamics as well (31, 32). Therefore, a holistic evaluation of complex herring ecology, human impacts, and the dynamic Baltic ecosystem is merited.

## The Baltic Ecosystem

The Baltic exhibits a stark salinity gradient, from near-ocean levels where it connects with the Kattegat to near-freshwater levels in the gulfs (33). Baltic herring is a schooling fish with enormous population sizes, with current stock estimates on the order of millions of tons of spawning stock biomass (7). Herring show strong genetic adaptation for spawning season and adaptation to differing salinity conditions (23, 34–37), ultimately segregating into two distinct metapopulations: spring spawners and autumn spawners (38, 39). Spring spawners are smaller and mature more quickly than autumn spawners, and prefer to spawn in coastal areas as opposed to the deeper waters used by the slow-growing, larger autumn spawners (30, 40). Given the larger size of autumn-spawning herring, we expect that earlier fishing efforts prioritized these populations over the smaller spring-spawning aggregations. Each metapopulation is further separated into management stocks living in the western Baltic (ICES subdivisions 21 to 24), central Baltic (ICES subdivisions 25 to 27, 28.2, and 29), and gulfs (ICES subdivisions 28.1 and 30 to 32) (Fig. 1*A*) (30, 41). The western Baltic autumn-spawning stock was the population targeted by the Øresund fishery. Western Baltic herring (both spring and autumn spawners) spawn in coastal areas of the Kattegat and southwestern Baltic (31, 42), and are likely reproductively isolated populations from the rest of the Baltic (43, 44).

Herring remain an important cultural and commercial industry in the Baltic (7). As Europe attempts to shift toward a more “sustainable” diet based on aquaculture and marine resources, herring are becoming a key species in the proposed fight to reduce global carbon footprints (45–49). Baltic herring are facing an additional challenge in our attempts to reduce land-based meat consumption both through a regional increase in fishmeal production for aquaculture (50) as well as direct consumption. In the European Union, there have been calls to increase understanding of ocean ecosystems to assess the viability of feeding the world from the ocean (48). Ecologists have already noted the disconnect between the reality of modern ocean environments and governments’ proposed sustainable new marine industries (49), although it is unclear whether this analysis holds true in all ecosystems or over time.

Here, we publish a long time series of genome-wide aDNA data for Baltic herring, analyzing 40 archaeological herring specimens from seven sites in Poland, Denmark, and Estonia that span the period from 750 to 1600 CE. Using *BAMscorer*, software specifically designed for population assignment using ultra-low-coverage sequence data (51), we identify the biological origin of each specimen, illustrating the change in fishery targets throughout time and evaluating the onset of long-range fish trade in the Baltic. We use modern whole-genome resequencing data—including 53 sequences generated for this study—to investigate population structure and model recent past demography for each of the Baltic herring populations in our data (Fig. 1*C*). We assess the difference in western Baltic autumn-spawning herring outcomes for the Øresund fishery in comparison with more recent fishing efforts and provide insight into the timing of herring population turnover in the Baltic. We compare the demographic trajectories of four Baltic herring populations with known historical events, including changed fishing practices and temporal changes in sea surface temperature, to assess the impact of these events on herring population size and the long-term sustainability of this iconic industry.

## Results

### Population Structure.

We resolved modern population structure in a principal-component analysis (PCA) based on 10 million genome-wide single-nucleotide polymorphisms (SNPs) in 68 modern individuals (Fig. 1 *A* and *B*), consistently separating populations based on geography (Baltic or Atlantic) and spawning season (Fig. 1*C*). We further observed fine-scaled population structure within spawning ecotypes in the Baltic, with central Baltic and gulf spring spawners, as well as central Baltic and western Baltic autumn spawners, clustering closer to their respective sample locations (Fig. 1*C*). This fine-scaled structure was also supported by higher levels of relatedness within each sample location and subsequently lowered levels of relatedness in these subpopulations when grouped as metapopulations (*SI Appendix*, *Text* and Fig. S9). The modern mitogenomes revealed three major clades [IQ-TREE (52, 53)] that exhibited no association with geography or environment across the Baltic and Atlantic, in accordance with previously published results (44). All archaeological mitogenomes clustered with the modern Atlantic and Baltic samples, identifying these as herring (*SI Appendix*, Fig. S11). Genetic diversity [π; VCFtools v0.1.16 (54)] across the genome was significantly higher for autumn-spawning herring (*SI Appendix*, Fig. S12). This higher diversity indicates a larger effective population size over time for the autumn spawners relative to spring spawners.

### Population Assignment.

**Fig. 2.**
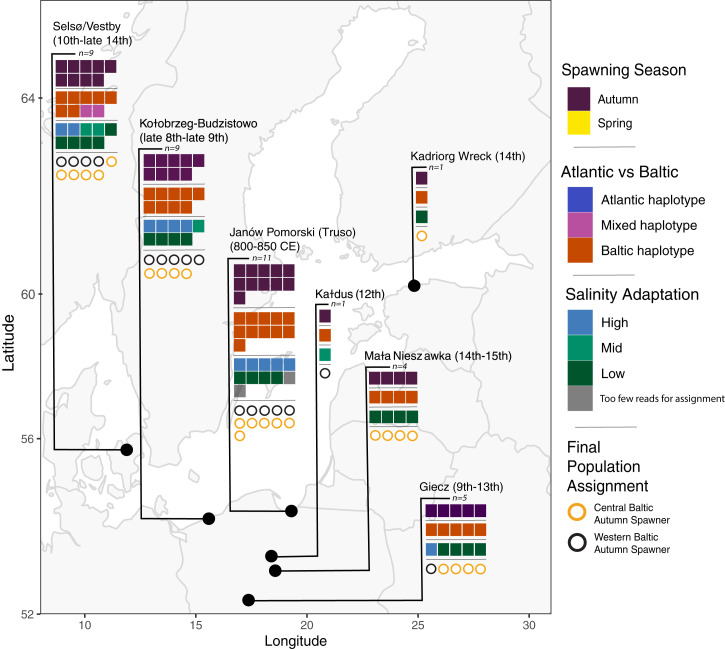
Population assignment results for archaeological herring specimens. Each individual is represented by a single square for each assignment test. The three assignment tests (spawning season, chromosome 12 inversion type for autumn-spawning specimens, and salinity adaptation) are then combined to make a final population assignment illustrated by the row of circles for each site. Archaeological specimens were assigned as autumn- or spring-spawning across sites reported to be associated spawning seasons by Han et al. (37). Atlantic and Baltic chromosome 12 inversion types were assigned based on *BAMscorer* sensitivity analysis (*Materials and Methods* and *SI Appendix*, Fig. S7). Salinity was assigned using SNPs reported by Han et al. (37) to be associated with adaptation to salinity. Some archaeological specimens had assignment values in between high and low (Dataset S1), and therefore were classified as stemming from “mid”-level salinity. Western Baltic specimens are present in the earlier sites from around Poland whereas later sites and contexts trend toward central Baltic specimens. Context-specific results can be found in Dataset S1.

All 40 archaeological samples were classified using *BAMscorer* as autumn-spawning herring. Autumn-spawning Baltic and Atlantic herring are genetically differentiated at the chromosome 12 inversion (37). All specimens could therefore be assigned to either the Atlantic or the Baltic using their inversion haplotype. Nearly all exhibited the chromosome 12 inversion haplotype (BB) associated with Baltic herring (Fig. 2). Spawning season and chromosome 12 inversion type could be assigned with as few as 50,000 reads, while salinity adaptation required 60,000 reads for accurate assignment (*SI Appendix*, Figs. S6–S8). Two specimens from Truso had too few reads to properly assign a salinity adaptation but could be assigned for chromosome 12 inversion type and spawning season (Fig. 2 and Dataset S2).

Salinity adaptation was mixed between all sites, with earlier sites showing some individuals with higher salinity adaptation, which is indicative of populations that spend part of their annual cycle in the Skagerrak and North Sea, such as the western Baltic autumn-spawning herring, and later sites showing more low salinity–adapted individuals (Fig. 2 and Dataset S2). Indeed, the oldest samples in the dataset—from Truso in Poland, dating to 800 to 850 CE (21)—showed nearly half of samples stemming from high salinity–adapted populations. In contrast, the sites dating to later periods were more dominated by low salinity–adapted autumn spawners (Table 1). This change indicates a potential spatial shift in target population for Baltic fisheries over time from western to central Baltic, although further studies with larger sample sizes are merited to confirm this conclusion.

**Table 1.** Percentage of WBAS herring specimens present in sampled specimens from each archaeological assemblage

Site	Date	WBAS, %	*n*
Janów Pomorski (Truso)	800 to 850 CE	45.5	11
Kołobrzeg-Budzistowo	Late 8th to late 9th century	55.6	9
Giecz	9th to 13th century	20	5
Selsø/Vestby	10th to late 14th century	44.4	9
Kałdus	12th century	100	1
Kadriorg wreck	14th century	0	1
Mała Nieszawka	14th to 15th century	0	4

### Demographic Analysis.

Both Baltic autumn-spawning populations showed fewer and shorter runs of homozygosity (ROHs) across all time bins (*SI Appendix*, Fig. S13). This pattern indicates either a large effective population size or population growth (55). The Baltic spring-spawning populations exhibited signs of smaller effective population size and/or population decline across all time bins. No ROHs >330 kb were found in any population, likely due to the high recombination rate in herring (2.54 cM/Mb) (36) and small sample size for this analysis. The combined ROH for all time periods indicates a large effective population size in the past for autumn-spawning herring and smaller effective population size over time for spring-spawning herring.

**Fig. 3.**
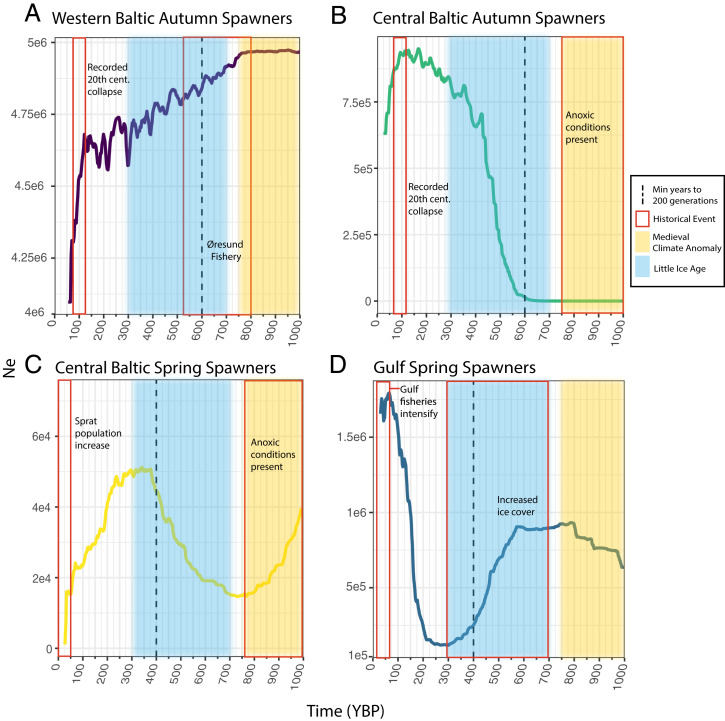
Temporal reconstruction of recent effective population sizes in four Baltic herring stocks. Each stock is represented in a separate chart: WBAS, CBAS, CBSS, and GSS. The *y* axis indicates estimated *N*_e_ in the past while the *x* axis indicates time in years before present. Colored rectangles indicate key historical events. Yellow rectangles show the approximate duration of the MCA and blue rectangles indicate the LIA. The dashed vertical lines show the minimum date (YBP) at which 200 generations in the past is reached (calculated using minimum generation times of 3 y for autumn spawners and 2 y for spring spawners), the known accurate window for *gone*. Historical events are denoted by red boxes for each population. (*A*) Demographic trajectory of the western autumn spawners shows a decline starting shortly after the start of the Øresund herring. They show an additional severe decline corresponding to the reported 20th-century autumn-spawning fishery collapse in the Baltic. Please note that, for reasons of scale, the *y* axis does not start at zero for this panel. (*B*) Demographic trajectory of central autumn spawners, which appear limited during the MCA when anoxic conditions are present in the central Baltic. They increase during the LIA and rapidly decline during the period of known autumn-spawning population collapse coinciding with the increase of the sprat (*S. sprattus*) population. (*C*) Central spring spawners show an increase around the time of the decline of the western autumn spawners, and then a decrease again at the end of the LIA as well as another dramatic decrease around the time of the autumn spawners’ collapse ∼100 YBP. (*D*) GSS decreases during the LIA and then increases dramatically at the end of the LIA, starting to decline only in very recent generations when fisheries in the gulfs intensify. Please note that, for reasons of scale, the *y* axis does not start at zero for this panel.

Highly divergent demographic trajectories are obtained using *gone* (56) for each of the four Baltic populations under study (Fig. 3): central Baltic spring spawners (CBSS), central Baltic autumn spawners (CBAS), western Baltic autumn spawners (WBAS), and gulf spring spawners (GSS). These results illustrate long-term demographic independence of the four main herring stocks in the Baltic. The population size of WBAS started declining ∼800 y before present (YBP), which corresponds to the onset of the Øresund fishery (Fig. 3). The WBAS population never fully recovered from this decline, gradually dropping off until ∼100 to 150 y ago, at which point the decline became much more rapid. In contrast, the CBAS population exhibited a strong population increase ∼600 YBP and continued to climb until ∼100 YBP, at which point they rapidly declined. Coincident with the initial decline of WBAS, the central Baltic spring-spawning herring (CBSS) began to increase. They plateaued around 500 YBP and then started to decline ∼400 YBP. Gulf Spring-spawning herring (GSS) showed a strong population increase until very recently, only showing a decline in recent generations. Each population is plotted with relevant historical events noted, including the onset of particular fisheries as well as the duration of two key past climatic events: the MCA and the LIA, during which the Baltic was, respectively, warmer and much colder than today, albeit with short-term variability (32, 57).

## Discussion

Using modern and historical whole-genome sequences from Atlantic and Baltic herring, we resolve herring population structure, reveal the earliest known large-distance herring trade, and propose that historical fishing operations had an observable genetic impact. We here contextualize our results with historical, ecological, and archaeological perspectives to situate our findings in a wider narrative of human–ocean interaction.

### Population Assignment.

We find no evidence of spring spawners in the archaeological specimens analyzed. There are multiple reasons this might be the case. Historical evidence shows that the medieval fisheries, including the Øresund fishery, were often strictly regulated to target spawning aggregations, and thus a single herring stock (16, 27). Further, when population sizes of the autumn-spawning herring were high, it is likely that the spring-spawning herring were low; data from other oceanic basins show coexistence of the two metapopulations, but that one seasonal spawner appears to dominate (24, 25). All pre–20th century Baltic fisheries under consideration appear to have focused on autumn-spawning herring. This observation provides genetic evidence for historical arguments that autumn spawners were the dominant metapopulation in the Baltic in the Middle Ages. We further show that both past and present Baltic fisheries focused on Baltic herring rather than Atlantic herring as previously supposed (8).

Our archaeological specimens exhibited variation in adaptation to salinity. This result reflects the differentiation between herring that spawned within the Baltic Proper and those that spawned within the transition zone of the Kattegat and western Baltic (37). The higher salinity–adapted individuals appear in multiple sites in Poland, which would not have had high salinity–adapted communities spawning near the coast. While salinity conditions in the Baltic have changed slightly in the last 1,000 y (58), the changes were not significant enough that ocean-level salinity conditions would be present in the central Baltic. Strikingly, our samples from Truso, which date between 800 and 850 CE (21), show the presence of high salinity–adapted herring with Baltic-type inversions on chromosome 12 which could only stem from fishing operations in the Kattegat or western Baltic (37). Previous studies have shown that while there is currently a small degree of connectivity between the central and western Baltic, the majority of this is unidirectional toward the western Baltic (44). Furthermore, populations from the western Baltic have been shown to be genetically distinct from the central Baltic (43), and therefore it is highly unlikely that these herring were fished locally. Assemblages with later dates show a shift toward lower representation of high salinity–adapted herring (Fig. 2 and Table 1). This observation reflects the known historical decline of the Øresund fishery, and shows the existence of refugia for the Baltic autumn-spawning herring metapopulation during the Medieval Warm Period, when anoxic conditions were prevalent in the central Baltic (Fig. 3).

The discovery of high salinity–adapted herring at Truso provides genetic evidence that this assemblage stemmed from a product of trade, a hypothesis suggested by the type of bones retrieved (16). With an origin stemming from the western Baltic region, these fish thus show the existence of long-distance fish trade in the Baltic at an earlier date than previously believed. This observation expands the scale of earlier observations of long-distance cod trade during the Viking Age (59) and its scope, given that herring trade is technologically more complex (requiring salting rather than only drying) (5).

### Demographic Analysis.

Demographic reconstruction showed distinct trajectories for each of the four populations under consideration. These reconstructions highlight the importance of management based on biological units, and indicate the differential effects of fishing pressure and climate change on specific populations of the same species within the same region. We here evaluate possible varying impacts of fishing pressure and climatic change (e.g., sea surface temperature) on each of the four stocks in turn to illustrate this point. We here illustrate how these population stock dynamics are not always consistent with expected change due to climate impacts alone.

### Western Baltic Autumn Spawners.

WBAS exhibits a population decline coincident with the intensification of fishing in the Øresund region. High temperatures in the central Baltic have a limiting effect on autumn-spawning herring reproduction (41). We would therefore expect the end of the MCA (1,000 to 800 YBP) to result in increased population size for WBAS in the absence of fishing pressure. In contrast, WBAS begin to decline ∼800 YBP and continued to decline until the present day. We observe a small drop corresponding to the proposed collapse in the 16th century described by Lehmann et al. (16), yet the population did not suffer a drastic contraction until ∼100 YBP. We conclude that the 16th-century decline of the Øresund fishery was therefore not due to a complete population collapse, although the fishery clearly had a significant impact on the overall size of the WBAS stock.

### Central Baltic Autumn Spawners.

CBAS exhibits extremely low effective population size until the onset of the LIA (∼700 YBP), at which point it increases dramatically until ∼100 YBP. Sediment cores have shown that temperatures in the central Baltic during the MCA were warm enough to produce algal blooms and anoxic conditions (32, 58, 60). Autumn-spawning herring spawn in deeper waters than spring spawners, and are therefore more vulnerable to anoxic conditions (61). Combined with temperature-limited reproductive success (41), CBAS was thus likely under limiting environmental conditions prior to the LIA. Our results show an expansion of this population that mirrors the timing of the WBAS decline and the onset of the LIA, a time when there would also have been low fishing pressure in the central Baltic. Subsequently, we see a dramatic reduction in CBAS effective population size corresponding to the historically documented 20th-century decline of autumn-spawning fisheries (26, 29). This decline has previously been shown to be caused by overfishing rather than direct or indirect climate impacts, including warming temperatures and eutrophication (30).

### Central Baltic Spring Spawners.

CBSS shows dramatic changes in effective population size over time. We expected to see an increase in the CBSS population concurrent with the autumn-spawning decline ∼100 YBP, as proposed by the hypothesis that a demographic transition occurred in the Baltic, yet this is not reflected in the results. It is unclear why CBSS shows a decline at the end of the LIA, although this is perhaps in response to the growing CBAS population. One would expect that CBSS would fill the niche left by the collapsing CBAS population in the 20th century, yet it does not appear to do so. Interestingly, sprat (*Sprattus sprattus*) operate in the same niche as Baltic herring, and the two species have been shown to limit each other’s population sizes (62). Prior to the mid 20th century, sprat were rare in the fishing record in the Baltic (63), only appearing coincident with the decline of the CBAS herring. CBSS may therefore have been limited in taking over the niche left by the autumn spawners’ decline due to an increase in the sprat population around the same time (64). Thus, the domination of spring spawners in the 20th- and 21st-century fisheries may reflect not a population expansion of the spring spawners but a population collapse of WBAS and CBAS. Since the industrial fishery began targeting the CBSS ∼100 YBP, we see a significant reduction in effective population size. ICES reports also indicate a declining population over the last 50 y, with some variation in the past decade (65). There has not been a strong year-class for this stock since 2014 (66).

### Gulf Spring Spawners.

GSS has been subject to low fishing pressure throughout history (67), and therefore its demographic trajectory is likely more determined by changing environmental factors than the other herring stocks. Low temperatures and increased ice cover in the gulfs have been shown to have a limiting effect on gulf herring recruitment (40), and therefore a rise in population size after the LIA is to be expected and we see this in our demographic reconstructions. Fishing pressure on the gulf herring was not significant until the latter half of the 20th century (67). Our results follow this trend, in that GSS starts to decline in recent generations, but it is possible that demographic changes could also be due to climate change and increasing temperatures in the gulfs. It is likely too early to tell which factors are the driving components in the changed demographic trend for gulf herring in recent generations.

### Limitations.

While *gone* is stated as accurate for a minimum sample size of two individuals, it is possible that small sample sizes can affect the demographic calculations (56). The impact of small sample sizes is also reflected in the ROH analysis, which showed similar patterns to *gone* in that autumn spawners had fewer coalescent events in the past compared with spring spawners, indicating larger effective population size in the past. However, the ROH for CBAS lacked expected coalescent events that would reflect the low effective population size prior to 600 YBP (*SI Appendix*, Fig. S13). Where *gone* can be used with as few as two samples, ROHs generally require four to six (68), and therefore we advise caution in interpreting these results further than supporting the general trend of autumn-spawner dominance prior to 200 YBP.

Additionally, we offset each trajectory by minimum generation time for each stock due to the difficulty of accounting for changes in year-class strength over time. This is likely a conservative estimate, as herring are known to spawn in overlapping generations, and both local climatic conditions (69, 70) and fishing pressure (71) have been demonstrated to impact generation time and life history in herring. However, given the cyclical nature of year-class strength, it is not possible to accurately model changes in generation time. Additionally, the strength of particular year-classes has been shown to change drastically in response to changes in sea surface temperature and ice cover (69), meaning it is not always the larger, older fish that are contributing the most to the next generation and complicating determination of average generation time. Given this uncertainty, the minimum generation time is the lowest common denominator we can use, as this is the earliest age fish in these populations are known to reproduce and therefore can impact the next generation. Demographic reconstructions using longer generation times revealed similar impacts of climate change and exploitation pressure and are included in *SI Appendix*, Fig. S14.

Further, *gone* is known to be highly sensitive to population structure, and therefore connectivity between populations and/or the existence of substructure can affect results (56). Given the possibility of connectivity between GSS and CBSS (44), we further assessed the demographic trajectory of the Baltic spring spawners as a single metapopulation (*SI Appendix*, Fig. S15). This estimate shows a mixture of the results from the CBSS and GSS analyses, but is consistent with our conclusion that both changing temperatures and fishing pressure have impacted Baltic herring demography. The combined analysis also shows a decline similar to the GSS in recent generations.

Any study exploring effective population size (*N*_e_) reconstructions would be remiss not to include discussion of *N*_e_ and its complex relationship with census size (*N*). *N*_e_ is an estimate of levels of genetic diversity and capacity for genetic drift translated into a number that we often interpret as a proxy for population size (72). Thus, *N*_e_ is an important value for assessing the evolutionary health of a population, for example, estimating extinction risk. Reductions in *N*_e_ are indicative of events such as inbreeding depression, which means fewer adult individuals are contributing to the next generation (73, 74). Reductions in *N*_e_ can depress a population’s resilience to overexploitation and adaptive capacity, thereby impacting the evolutionary health of a population (75).

The relationship between *N*_e_ and *N* is complex, particularly for fish species such as herring with enormous census sizes and sweepstakes-style reproduction strategies (76). Further, population substructure and differences in productivity can result in drastically different *N*_e_/*N* ratios (77). As each of the herring populations here is demonstrably independent, it is possible that the *N*_e_/*N* ratio is unique to each population. The facts that the *N*_e_/*N* ratio is here unknown and that *N*_e_ is a marker of demographic change are crucial distinctions to understand in the interpretation of this study. It is the relative changes for each population that indicate the impact of exploitation and climate change rather than the absolute estimates of *N*_e_. We show that herring stocks exhibit differential responses to both climate changes and fishing pressure, each of which has an evolutionary impact on the species. Evolutionary risk is not often applied to conservation and fisheries management policies, but should be taken into account to maximize stock and species health (75).

### Human and Environmental Impacts on the Baltic Herring.

We here present observations that are consistent with early human fisheries in the Baltic having an impact on herring population trajectories. To address growing demand for marine fish protein in Europe, the initial fishery (beginning by 850 CE and expanding especially ∼1200 CE) focused on the most economically relevant herring with the biggest spawning aggregations, the western Baltic autumn-spawning herring. The onset of this fishery corresponded to a negative population trajectory at a time when environmental conditions for the stock were improving. Fishing finally ceased in Øresund during the 1600s, which may have been associated with changes in stock size due to overfishing. Fishing pressure then focused on the next-largest herring stock, CBAS, and the pressure again intensified until it collapsed. After the highly valued autumn spawners were fully exhausted in the early 20th century, only then did the herring fishery turn to targeting spring spawners, the smaller, less-valued population. And finally, GSS, long disregarded as a potential commercial target, is now facing the pressures of industrial fishing (78–80).

The obtained demographic trajectories cannot be explained by changes in climate alone. Rather, they indicate that each stock has experienced trajectories in the past (or currently) that do not correspond to changes in climate. Moreover, for CBAS, the demographic trends reported here in the last 150 y can be directly attributable to fishing pressure rather than climate (30).

Baltic herring are currently facing the combined challenges of overfishing, eutrophication, rising temperatures, competition from expanding sprat populations, and further dilution of the Baltic by fresh water (10, 18, 24, 62, 81–83). In recent years, ICES has recommended an MSY of 0 for several Baltic herring stocks (7, 78, 84, 85). Yet, fishing continues and the stocks do not recover (86), because economic and cultural factors continue to drive exploitation. In the face of these issues, it may seem that the fate of the herring is sealed. However, modeling of Baltic ecosystem dynamics has shown that fisheries policy and conservation management are some of the most important factors in determining the long-term sustainability of the Baltic (10, 87). Therefore, it is crucial that we learn from the past, both recent and further-reaching, to better understand the real impact of various management and extraction policies on the demography of Baltic herring throughout time, as well as the differential responses of each herring stock to varying environmental pressures.

By bringing historical ecology methods into play, we here elucidate ecological and cultural thresholds (88) that were crossed as the Baltic fisheries changed over time, finally resulting in the Baltic we see today. These thresholds—including cultural change [e.g., increased demand for marine fish because of rising urbanism and religious requirements (2)], ecosystem change, and shifting exploitation patterns—can be used to inform management policies in the future by reframing what constitutes healthy population dynamics (size, migratory behavior, etc.) to better reflect long-term ecosystem dynamics. Further, the demonstrated demographic independence of each Baltic herring stock here assessed provides further support for managing each stock separately. Finally, our research highlights the interconnected nature of herring stock dynamics, climatic change, and fishing pressure. Given the challenges facing the Baltic due to ongoing climate change and exploitation pressure, it is crucial that management bodies take this interconnected feedback system into account.

The gulf spring-spawning herring are the last remaining healthy herring stocks in the Baltic (84, 89). They are also the stock most adapted to the brackish conditions which are expected to increase in the Baltic into the future. In order to preserve this stock, it is crucial that management bodies recognize this pattern of serial exploitation and climate impact in the Baltic, thereby providing avenues to identify specific exploitation strategies that have resulted in population collapse in the past (90) and avoid these for the future. Further, the differing responses to climate change as well as the evolutionary health for each stock should be considered when providing quotas and assessing stocks in the changing future. This could take the form of including genetic information such as effective population size in stock assessments rather than exclusively relying on estimated census size (75, 91)—which would entail further research on the relationship between *N*_e_ and *N* for the Baltic herring stocks—or assessing historical management strategies associated with fluctuations in stock size.

The demographic trajectories of CBAS and GSS provide further avenues of hope for the Baltic herring. Both stocks’ demographic reconstructions indicate that in the absence of industrial fishing pressure, herring stocks can rapidly recover from long-term exposure to poor environmental conditions. If the changing climate in the Baltic can be addressed sufficiently to maintain suitable threshold conditions for herring and fishing reduced in line with ICES recommendations, we may yet be able to preserve a significant spring-spawning Baltic herring population.

## Materials and Methods

### Archaeological Material and Laboratory Methods.

Archaeological bone samples were obtained from seven sites in Poland, Denmark, and Estonia spanning 800 to 1600 CE (Fig. 1*A*). A full sampling table with site information can be found in Dataset S1. Samples were processed following the laboratory pipeline in Ferrari and Atmore et al. (51). Full laboratory methods can be found in *SI Appendix*, with library protocols detailed in Dataset S1. All laboratory protocols were carried out in the dedicated aDNA laboratory at the University of Oslo following regular decontamination and authentication protocols (92–94). Samples yielded 0.002 to 2.5× coverage.

### Modern Genome Sampling.

Fifty-three tissue samples were collected around the Baltic and the Norwegian coast between 2002 and 2010 as reported in Ruzzante et al. (95) and André et al. (96) (Fig. 1*B*). DNA was extracted from the tissue samples using a DNeasy Blood and Tissue Kit. Library building and sequencing were carried out at the Norwegian Sequence Centre. Samples were sequenced on an Illumina HiSeq 4000, yielding coverage of 7 to 17×. An additional 22 individual whole-genome sequences were obtained from previously published data to ensure that all major herring populations were represented (37). Coverage from publicly available sequences ranged from ∼15 to 55×. Full metadata for all modern samples can be found in Dataset S2.

### Alignment and SNP Calling.

Both modern and ancient sequences were aligned to herring reference genome Ch_v2.0.2 (36). All sequences were aligned using PALEOMIX v1.2.13 (97). Modern sequences were aligned using bwa-mem and ancient sequences were aligned using bwa-aln. mapDamage2.0 (98) plots for postmortem deamination were assessed to validate the ancient samples (*SI Appendix*, Fig. S1). Modern nuclear sequences were further processed following the GATK best practices pipeline with GATK4 (99). Full methods are described in *SI Appendix*. Several individuals from the modern dataset were removed due to suspected contamination and/or incorrect metadata from the publicly available dataset. The final modern dataset comprised 52 genomes sequenced for this study and 16 publicly accessed genomes. The process of data cleaning and individual assessment is detailed in *SI Appendix*, Figs. S2–S4.

### Determining Population Structure.

smartPCA (100, 101) (Eigensoft v7.2.1) was run on the entire modern nuclear sequence dataset to assess population structure. A maximum-likelihood phylogenetic tree was then built with IQ-TREE v1.6.12 (52, 53) using the mitogenome dataset including all modern and ancient samples to verify that the archaeological samples are Atlantic or Baltic herring. A Pacific herring (*Clupea pallasii*) mitogenome—obtained from Han et al. (37)—was used as an outgroup.

Genetic diversity (π) was estimated in 100-kb windows along the nuclear genome using VCFtools v0.1.16 (54) for each of the four Baltic populations: WBASs, CBASs, CBSSs, and GSSs, with two or three samples representing each population. Samples were chosen randomly to control for differences in sample sizes between populations, and the same subsets were used for ROH analysis (see below). Full metadata for modern sequences can be found in Dataset S2. Fine-scaled population structure was determined using KING v1 (102), which calculates individual pairwise kinship coefficients within each population. Baltic spring spawners and Baltic autumn spawners were assessed as metapopulations to determine the level of relatedness between possible subgroups (e.g., gulf vs. central Baltic spring spawners and western vs. central Baltic autumn spawners).

### Population Assignment.

*BAMscorer* is capable of accurate genomic assignments using extremely low coverage data (51). Previous studies have reported that population structure in herring is driven by spawning season and adaptation to different levels of salinity (23, 34–37). Three sets of assignment tests were designed for *BAMscorer* using the following biological characteristics: spawning season, chromosome 12 inversion type (Atlantic vs. Baltic), and adaptation to salinity. These biological characteristics correspond to diagnostic genomic Atlantic herring population data (34, 36, 37), which shows strong genetic differentiation between spawning seasons (35, 37) and adaptation to salinity levels (23, 37), as well as differentiation between Baltic and Atlantic autumn-spawning herring at the chromosome 12 inversion (37).

To determine spawning season and salinity adaptation, we used databases of previously published diagnostic loci (37), containing 835 SNPs associated with spawning season and 2,303 SNPs associated with salinity. To determine the chromosome 12 inversion type, a database with 4,503 SNPs was created using the default parameters from the *BAMscorer* v1.4 *select_snps* module. We then performed a sensitivity analysis, following the methods from Ferrari and Atmore et al. (51), to assess the minimum required read depth for 100% classification of each assignment test. For this, we used whole-genome data of eight modern individuals with known metadata. These eight modern individuals were not included among those individuals used to obtain the 4,503 divergent SNPs. The aligned reads of those eight individuals were then randomly down-sampled to between 500 and 100k reads in increments of 500 reads up to 10,000 reads and then increments of 10,000 reads to simulate extreme low coverage data. For each increment and individual, the data were randomly bootstrapped (20 iterations). Assignment sensitivity was then assessed by running the assignment test on the down-sampled alignment files. Minimum required read depth was determined when all 20 randomly down-sampled files per individual were assigned correctly. Sensitivity tests were performed for each of the three assignment tests using the eight modern specimens with known metadata as test data. Full methods and results of assignment sensitivity analysis can be found in *SI Appendix*. After determining assignment sensitivity and required read depth, each test was then applied to those ancient individuals for which sufficient reads were obtained for accurate assignment. Only autumn-spawning Baltic and Atlantic herring are differentiated on the chromosome 12 inversion type. Therefore, a hierarchical approach was applied, first assessing spawning season, then chromosome 12 inversion, and finally salinity adaptation. These were then combined to yield a final population assignment.

### Demographic Reconstruction.

ROHs were analyzed per population to determine the general trends in effective population size for each population (complete methods can be found in *SI Appendix*). The timing of the coalescent events associated with specific windows of ROH length was determined using the formula *L* = 100/2*g* cM (103), where *L* refers to the length and *g* is generation time (*SI Appendix*, Fig. S11). ROHs were subsequently binned into two groups corresponding to 650 to 400 YBP and 400 to 200 YBP. We compared the total sum and number of ROHs for each bin as well as across all bins.

The four Baltic datasets containing 2 to 10 individuals were input into the demographic software package *gone* (56), which estimates *N*_e_ in the recent past using linkage disequilibrium (LD) decay. *N*_e_ estimates are geometric means taken from 40 bootstrapping iterations that randomly sample 50,000 SNPs from each chromosome in each population to estimate LD decay. *gone* has been shown to be robust to natural selection (104) and viable for small sample sizes (56). No minor-allele frequency filter was applied to the dataset used for *gone*. Default parameters were used except for substituting the known population-wide recombination rate for Baltic herring of 2.54 cM/Mb (36). *gone* results were scaled by generation using the minimum generation time in herring [3 y for autumn spawners and 2 y for spring spawners (105)]. Each population trajectory was additionally offset by the year the sampling took place (1979 to 2016; Dataset S2). As previous studies have suggested some connectivity between GSSs and CBSSs (44), an additional analysis was undertaken using the Baltic spring spawners as a single population (*SI Appendix*, Fig. S15).

We reconstructed population demography using *gone* v1, which is found to be accurate up to at least 200 generations in the past (56). We show trajectories up to 1,000 YBP to reflect the possibility of overlapping and/or longer generation times. As herring spawn in overlapping generations with varying maturation times, and can live up to 18 y (106), generation times in 5- and 10-y increments are further shown in *SI Appendix*, Fig. S14 to illustrate the possible variations in trajectory for each population. Exact *N*_e_ estimates of *gone* are likely affected by large effective population sizes in the past and the small sample size used here (56). True *N*_e_ estimation is further confounded by fish spawning behaviors, including overlapping generations and batch spawning (107). Therefore, the key findings from the *gone* analysis are the divergent trajectories and their relative changes rather than the absolute values of *N*_e_ estimated for each population at a given date. All four populations showed unrealistic bottlenecks to near-zero *N*_e_ in the four most recent generations. The original *gone* paper reports similar patterns for recent generations when using small sample sizes (*n* ≤ 10) and applications to other fish populations show a similar pattern (56, 107), and therefore these recent extreme bottlenecks were disregarded as an artifact of the calculations and removed from the results. Given the possibility of connectivity between the GSS and CBSS populations, *gone* was also used to estimate demographic history for the Baltic spring-spawning metapopulation as a whole (*SI Appendix*, Fig. S16).

## Supplementary Material

Supplementary File

Supplementary File

Supplementary File

## Data Availability

The raw genome sequence data reported in this article have been deposited in the European Nucleotide Archive (accession no. PRJEB52723) (108). All study data are included in the article and/or supporting information.
