# Supplementary material for: Population dynamics of Baltic herring since the Viking Age revealed by ancient DNA and genomics
Source: Proc Natl Acad Sci U S A. 2022 Oct 25;119(45):e2208703119. doi: 10.1073/pnas.2208703119 (PMC9659336; doi:10.1073/pnas.2208703119)
Supplement: Supplementary File [file pnas.2208703119.sapp.pdf]

Supplementary Information for

Population dynamics of Baltic herring since the Viking Age revealed by ancient DNA and genomics

Authors: Lane M. Atmore[1*], Lourdes Martínez-García[1], Daniel Makowiecki[2], Carl André[3], Lembi Lõugas[4], James H. Barrett[5*] & Bastiaan Star[1*]

Author affiliations:
1 Centre for Ecological and Evolutionary Synthesis, Department of Biosciences, University of Oslo, Oslo, Norway
2 Department of Environmental Archaeology and Human Paleoecology, Institute of Archaeology, Nicolaus Copernicus University, Toruń, Poland
3 Department of Marine Sciences – Tjärnö, University of Gothenburg, Strömstad, Sweden
4 Archaeological Research Collection, Tallinn University, Tallinn, Estonia
5 Department of Archaeology and Cultural History, NTNU University Museum, Norwegian University of Science and Technology, Trondheim, Norway
* Corresponding author

Correspondence:
Lane M. Atmore, lane@palaeome.org
James H. Barrett, james.barrett@ntnu.nu
Bastiaan Star, bastiaan.star@ibv.uio.no

This PDF file includes:

Supplementary text
Figures S1 to S15
Table S1
SI References

Other supplementary materials for this manuscript include the following:

Datasets S1 to S2

Supplementary Information Text

Full ancient DNA laboratory methods

Single-bone samples weighing 10-70mg were bathed in UV light to clean the exterior, then placed in digestion buffer (1 ml 0.5 M EDTA, 0.5 mg/ml proteinase K, 0.5% N-Lauryl sarcosine) and crushed in 1.5ml Eppendorf tubes using single-use plastic micro-pestles. DNA was extracted following the double-digest procedure from Damgaard et al.[1] with MinElute PB buffer (QIAGEN). Samples were then purified through MinElute columns using the QIAvac 24 Plus vacuum manifold system (QIAGEN) for a final elution volume of 65ul. Two types of libraries were built, with some samples built using the double-stranded library protocol from Meyer & Kircher[2] and some built with single-stranded libraries following the Santa Cruz protocol[3]. A full list of the library protocols used per sample can be found in Supplementary Dataset S1. All laboratory protocols were carried out in the dedicated ancient DNA laboratory at the University of Oslo following regular decontamination and authentication protocols[4–6]. Each library underwent 12-15 cycles of PCR amplification followed by purification using the Agencourt AMPure XP PCR purification kit using a 1:1 bead:sample ratio. Libraries were then assessed for quality on a Fragment Analyzer™ (Advanced Analytical) using the DNF-474 High Sensitivity Fragment Analysis Kit to determine suitability for sequencing. Samples of high enough quality were then sequenced on an Illumina HiSeq 4000 and/or NovaSeq 6000 at the Norwegian Sequencing Centre.

SNP calling and filtering

Modern samples were filtered using bcftools v1.3[7] (*FS<60.0 && SOR<4 && MQ>30.0 && QD > 2.0 && INFO/DP<415140' --SnpGap 10*) and VCFtools v0.1.16[8] (*--minGQ 15 --minDP 3 --remove-indels --maf 0.01*). Non-biallelic loci were removed. An additional dataset with no MAF frequency filtering was created for *gone* analyses known to be sensitive to removing minor alleles. Mitogenomes were called and filtered as above for ancient and modern samples. Individuals with >30% missingness were removed.

Removing outliers from the whole-genome dataset of modern herring specimens

Our dataset contains whole genome data of herring specimens obtained from a range of difference sources, collected over a number of years (see Supplementary Dataset S2). The dataset further combines publicly available data with data generated *de novo* for our study. Given this wide range of sources, we performed a number of exploratory analyses to ensure data integrity. First, we previously identified that two of the individuals from the Han et al.[9] dataset were technical duplicates[10] using KING[11] (Table S1). One of these individuals was chosen at random to be included in the dataset and the other was discarded. Second, exploratory population analyses using smartPCA[12,13] indicated that several individuals were significant outliers (Figure S2). To examine this pattern, levels of SNP-based levels of heterozygosity were calculated for the modern nuclear data using --het from VCFtools[8]. Three individuals (HER_Z12_IsleOfMan,

HER_NSSH34, and M-HER004) showed unusually high levels of heterozygosity (Figure S3), which is a measure of possible poor read mapping and contamination[16]. These individuals therefore show unusually high similarity in genetic information and/or poor read mapping yet high heterozygosity. This could be the result of cross-contamination between specimens. We further assessed the possibility of contamination by analyzing levels of heterozygosity along the mitogenome (Figure S4). As a haploid sequence, expected heterozygosity for each locus is 0. For those individuals containing loci with heterozygosity >0 this is indicative of contamination. Again, three individuals (HER_NSSH33, HER_NSSH34, and HER_Z12_IsleOfMan) showed clear signs of contamination. Based on these results, four individuals (HER_Z12_IsleOfMan, HER_NSSH33, HER_NSSH34, and M-HER004) were removed from the dataset. Finally, one individual (AAL1_CelticSea) from the Han et al. (2020) dataset was removed as it consistently clustered with individuals with non-matching metadata (Figure S5). Given other inconsistencies of the metadata, including the duplicated sample, this sample was also removed. The cleaned dataset was used for demographic (runs of homozygosity, KING, and *gone*) and for whole-genome PCA analyses.

BAMscorer sensitivity analysis

In order to determine which ancient sequences could reliably be scored, we first assessed the required read depth for accurate assignment in each of the three assignment tests (Chromosome 12 inversion, spawning season, and salinity adaptation). Required read depth per assignment was assessed following the down-sampling and bootstrap method of Ferrari & Atmore et al.[10]. We selected 8 samples as a training dataset from the modern reference dataset to independently test the power of assignment probability with known metadata. The alignment files of these eight samples were randomly down-sampled to between 1k and 100k reads 20 times and then used to assess BAMscorer sensitivity for each assignment test.

Given the relatively low number of autumn spawning herring, we investigated whether including the removed contaminated individuals (see above) impacted the biological patterns for each comparison. Although these outliers impact the whole genome population analyses, SmartPCA analysis on each of the three BAMscorer assignment databases showed that including these outliers associated with contamination bias did nothing to change the shape of the distributions. Due to limited sample size for the different categories in these assignment tests, several outliers were left in the BAMscorer databases apart from AAL1_CelticSea (suspected incorrect metadata) and Gavle54 (identical to Gavle98) from the Han et al.[9] dataset. Full information on which samples were used for each stage of analysis can be found in Supplementary Dataset S2.

The modern database showed strong differentiation between spring and autumn/winter spawning seasons, following previously reported results[9,17,18]. Sensitivity analysis showed that spawning season can be accurately determined in alignment files with as few as 50 000 reads (Fig S6). The chromosome 12

inversion could be confidently assigned with as few as 5 000 reads (Fig S7) using default parameters. Given that only samples for which spawning season could be assigned were analyzed, all samples still retained at least 50 000 reads. Salinity scores could be determined for samples with at least 60 000 reads (Fig S8).

Evaluating demographic independence

Demographic independence of the Baltic subpopulations was further assessed by calculating individual pairwise relatedness for each group with KING. To assess the substructure in the Baltic, metapopulations were grouped, resulting in two Baltic populations: autumn spawners and spring spawners (for full KING results, see Supplementary Dataset S2). In the Transition Zone (TZ) population, M-HER066 showed strongly negative kinship coefficients with the rest of the TZ individuals. This is a sign that there is population structure, thus M-HER066 is likely not actually a TZ individual, but could be part of the Atlantic spring spawners population, which is where it clusters on the PCA. M-HER066 was removed from analysis to eliminate outlier bias. Kinship coefficients were plotted per population to visualize the distribution of relatedness in each population (See Fig S9).

A one-way ANOVA showed that metapopulation ID was significantly associated with kinship coefficient mean and variance (p=8.16e-14, DF=4). Baltic spring spawners had the lowest average kinship coefficient (0.0336). Baltic autumn spawners had the second highest kinship coefficient (0.05827), likely due to the effect of the Fehmarn individuals, which showed high relatedness to each other. The Fehmarn within-group average was 0.08. This illustrates there is some substructure in the herring metapopulations, although it is likely that there is a degree of connectivity, as strong substructure would result in negative kinship coefficients. It should be noted that all individuals showed low levels of relatedness and there was some variance in relatedness between all groups. The Transition Zone showed the highest variation in kinship coefficient, likely due to the presence of subpopulations such as the Idefjørd herring.

Runs of Homozygosity

Initial ROH results showed that the variation in ROH length, count, and total sum were largely determined by differences in sample size. Therefore, a subset of each population was chosen randomly, with 2-3 individuals per population (samples used can be found in Supplementary Dataset S1). PLINK files were generated from the modern nuclear sequence data and then assessed for differences in runs of homozygosity (ROH) using PLINK 1.9[19] following previously-published recommendations[20–22]. The following command was used: *plink –bfile herring --chr-set 26 --double-id --homozyg-snp 50 –homozyg-kb 90 –homozyg-density 50 –homozyg-gap 1000 –homozyg-window-snp 50 –homozyg-window-het 3 –homozyg-window-missing 10 –homozyg-window-threshold 0.05 --out herring_roh.* ROHs were compared by length, count, and total sum between the 4 Baltic populations.

a

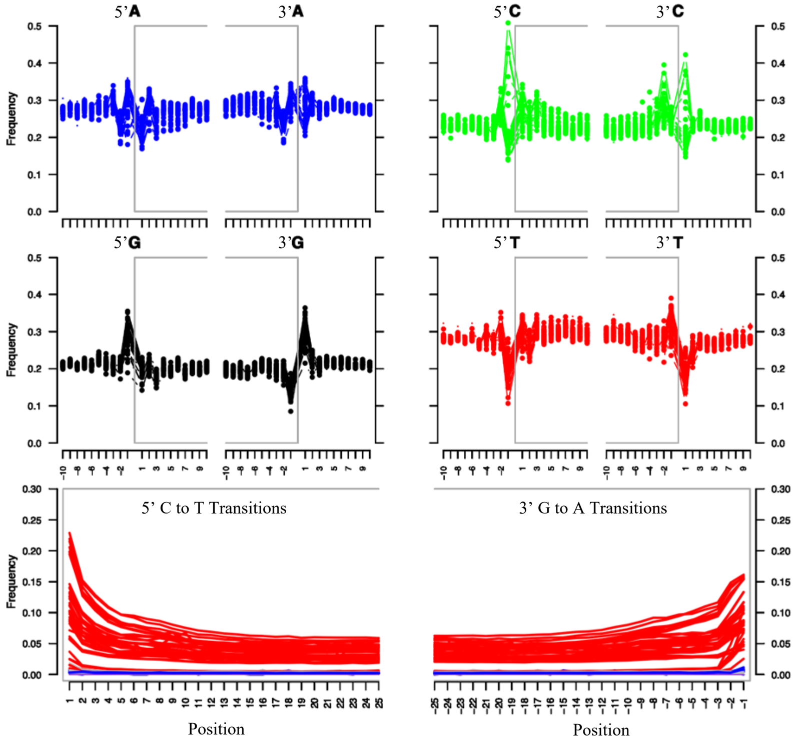
5'A
3'A
5'C
3'C
5'G
3'G
5'T
3'T
Frequency
5' C to T Transitions
3' G to A Transitions
Position
Position

b

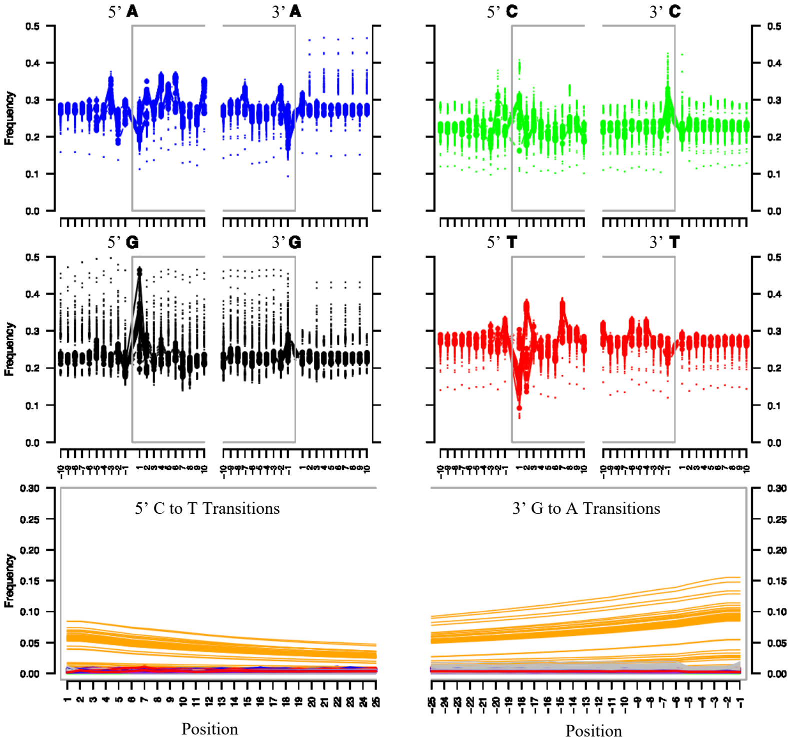
5' A
3' A
5' C
3' C
5' G
3' G
5' T
3' T
Frequency
5' C to T Transitions
3' G to A Transitions
Position
Position

Figure S1 – Fragment Misincorporation and ancient DNA Damage plots. a) Patterns were obtained by using MapDamage v. 2.0.6 after down-sampling BAM files to 1 million reads. These plots show the typical fragmentation and the post-mortem deamination patterns that are characteristic of those associated with ancient DNA[23]. We simultaneously plot all archaeological herring specimens (n=40) b) Fragment misincorporation and damage plots for modern herring sequence data (n=72).

Table S1 – KING output for technical duplicates/relatedness in modern herring reference data.
Concordance values above 0.8 are indicative of duplicates according to the KING documentation. Table from Ferrari et al[8]. Gavle54 and Gavle98 (Han et al.[9], PRJNA642736) are therefore considered technical duplicates. We removed Gavle54 from the dataset (see also Supplementary Dataset S2.

FID1	ID1	FID2	ID2	N	N_IBS0	N_IBS1	N_IBS2	Concord	HomConc	HetConc
**Gavle54**	Gavle54	Gavle98	Gavle98	11314407	187	4995	11309225	0.99954	0.99998	0.99674

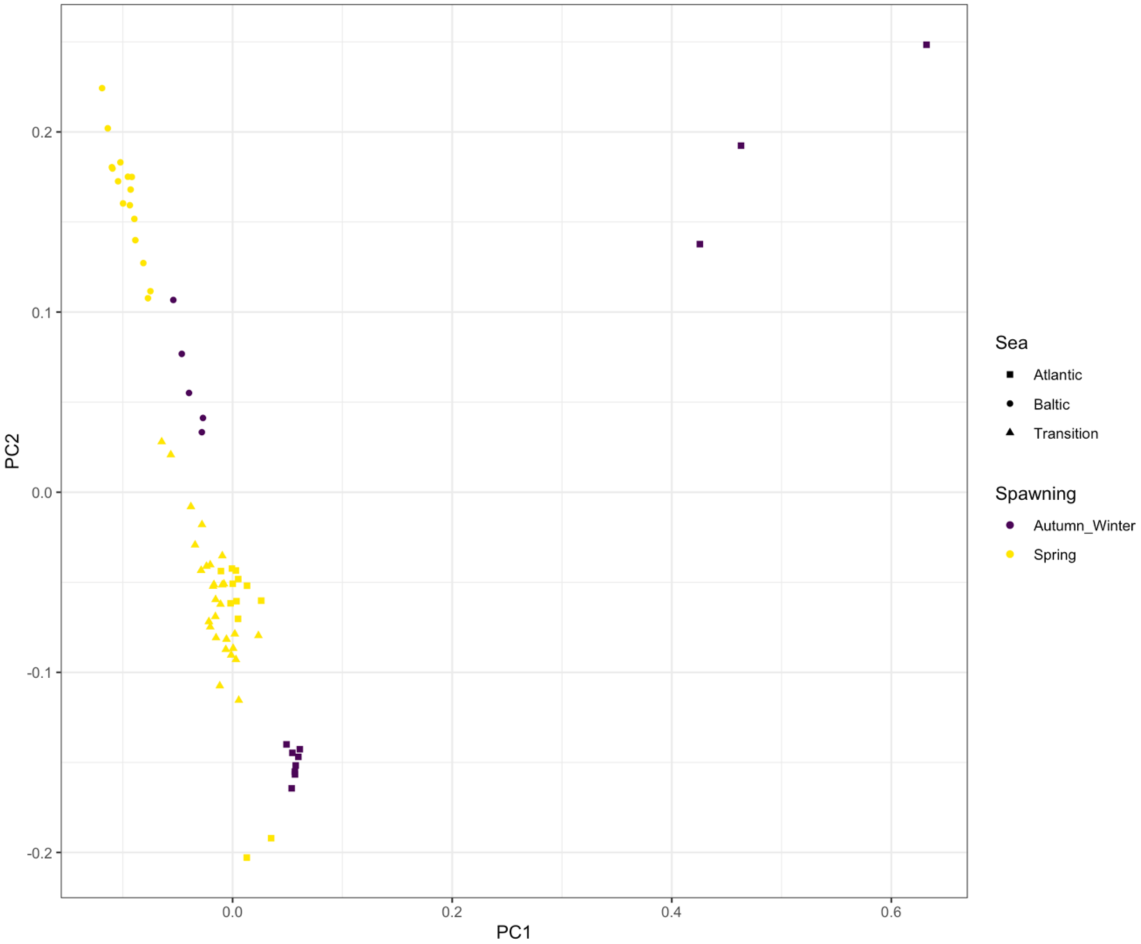

Figure S2 – Exploratory population analyses of Atlantic herring using genome-wide data. The PCA is based on 10,368,446 SNPs. The color indicates the spawning season and the shape indicates the sea in which the sample was collected. "Transition" refers to the area between Norway, Sweden, and Denmark that spans the transition between the North Sea and the Baltic Sea. Three Atlantic herring specimens are located away from the main herring clusters. This pattern is driven by inclusion of a single specimen (HER_Z12_IsleOfMan) that is contaminated (see also Figure S3, S4, and S5).

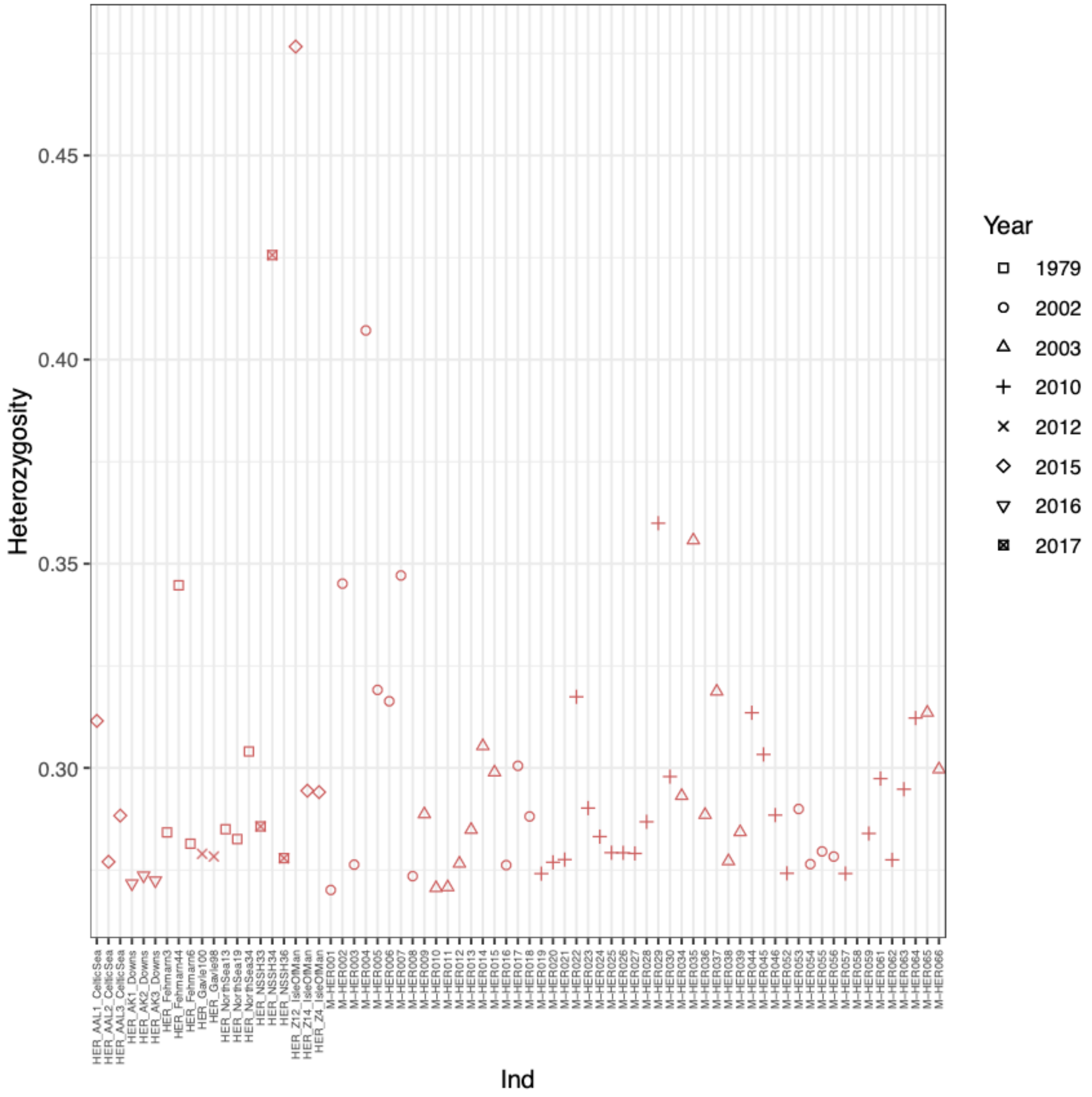

Figure S3 – Observed vs expected heterozygosity in modern herring specimens. Individual herring specimens are ordered along the x-axis. Shapes indicate the year in which the sample was obtained. Three individuals are clear outliers – HER_Z12_IsleOfMan, HER_NSSH34, and M-HER004.

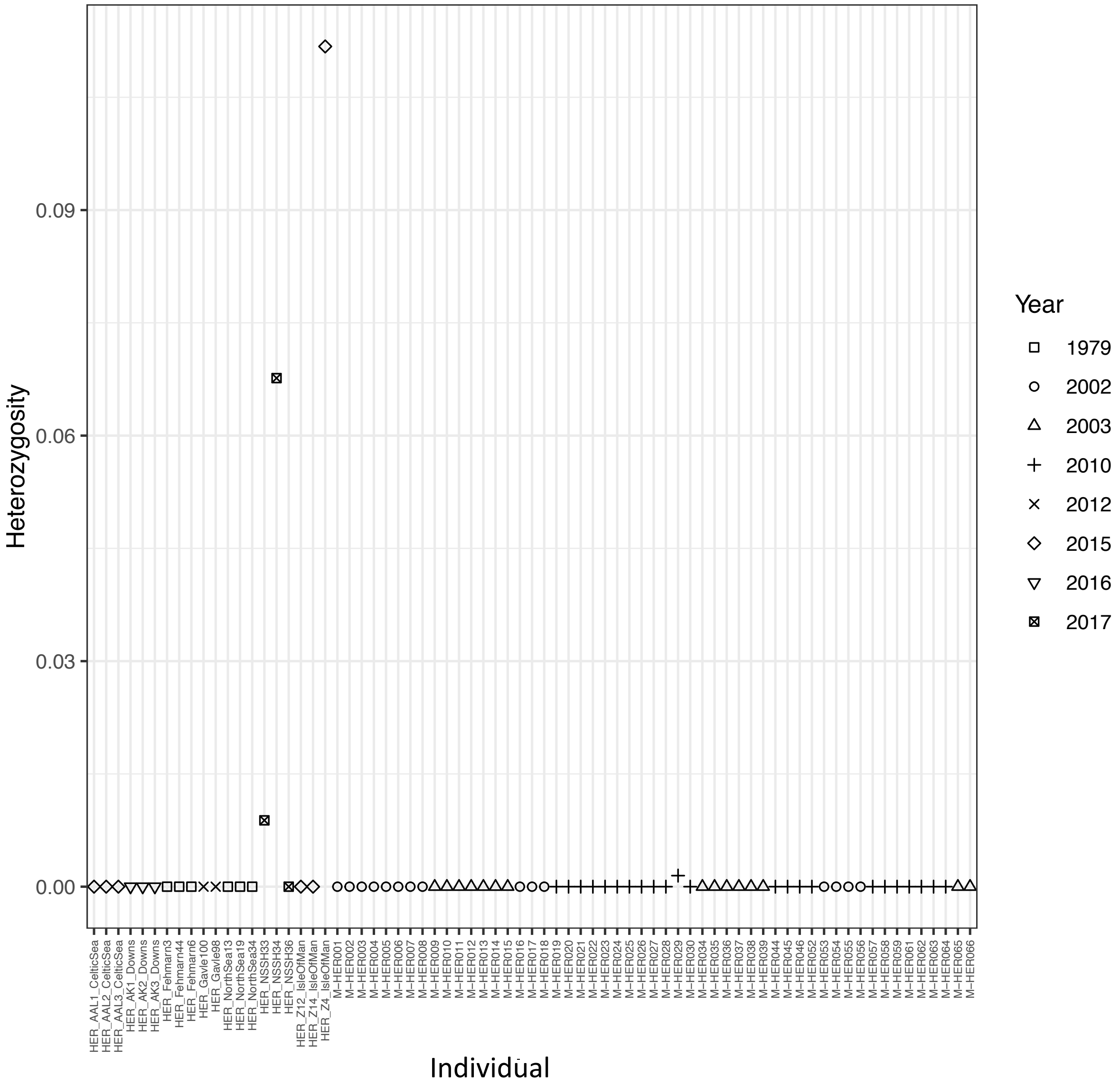

Figure S4 – Levels of heterozygosity observed in the mitogenome of modern herring specimens. SNPs were called on the mitogenome while allowing diploid genotype calls. Heterozygosity was calculated using VCFtools. We would expect the mitogenome to yield homozygote genotypes only. We observe three individuals with heterozygosity values >0, which is indicative of intraspecific contamination. These individuals are HER_NSSH33, HER_NSSH34, and HER_Z12_IsleOfMan. M-HER004 does not exhibit signs of contamination in the mitogenome, but was still removed from the dataset due to its high levels of heterozygosity.

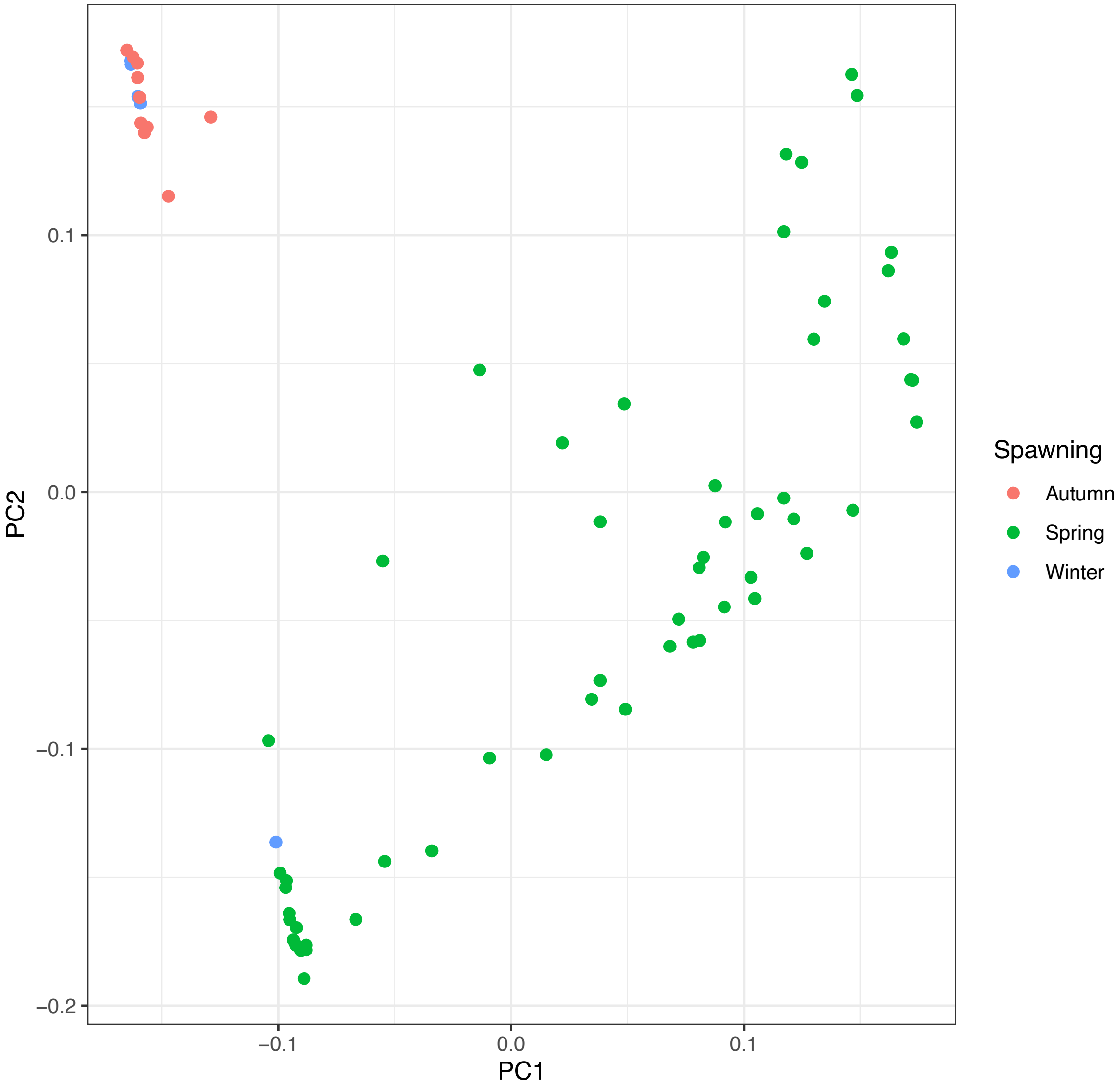

Figure S5 – PCA of spawning loci including all modern herring specimens. The PCA was run on whole genome data from 68 herring specimens (Supplementary Dataset S2) using a selection of spawning loci (n=835) that were obtained from Han et al[9]. We obtained a clear distinction between spring spawners and autumn/winter spawners. HER_AAL1_CelticSea from the Celtic Sea is coded as a winter spawner but consistently clustered with spring spawners in smartPCA analysis.

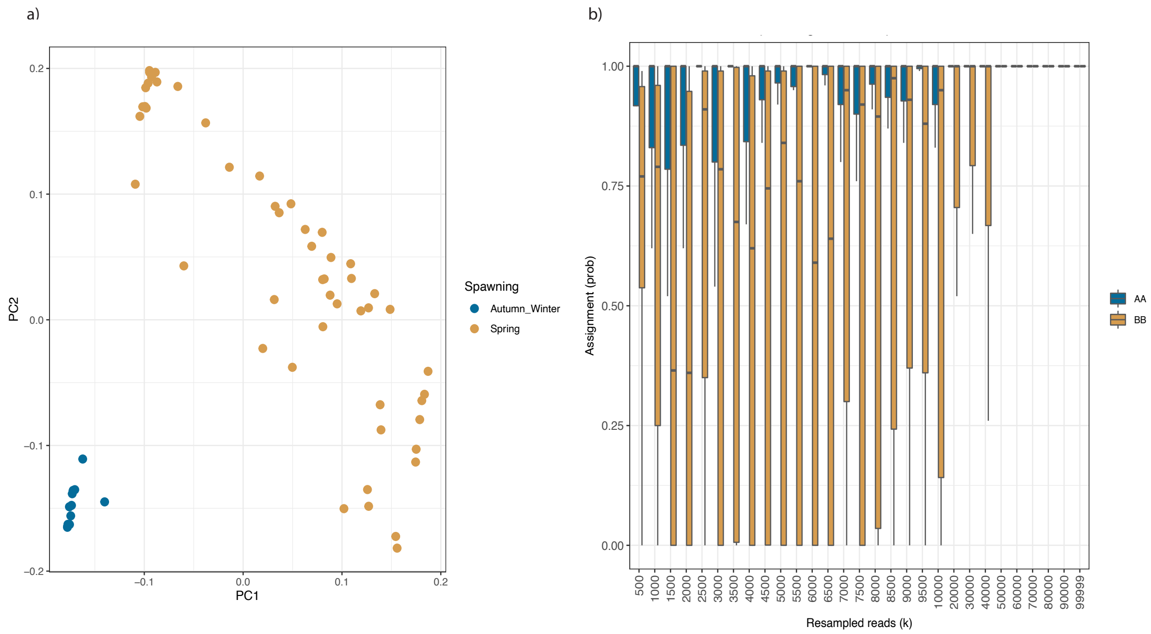

Figure S6 – Spawning season assignment sensitivity. a) PCA of the 835 diagnostic loci that identify spawning season from Han et al[9]. The PCA is based on whole genome data of 60 modern herring specimens (Supplementary Dataset S2), excluding eight modern herring specimens that were used as a test dataset with known metadata. b) Application of BAMscorer to the bootstrapped down-sampled alignment files of the eight test specimens with known metadata show greater difficulty assigning spring spawning than autumn spawning. Each individual BAM file from the training dataset was randomly down-sampled to between 500 and 100,000 reads for 20 iterations. The y-axis represents the proportion of those files which are known to be type AA or type BB that were assigned correctly using BAMscorer. Both seasons can be assigned with no error with a minimum of 50,000 reads. AA is autumn spawning and BB type is spring spawning.

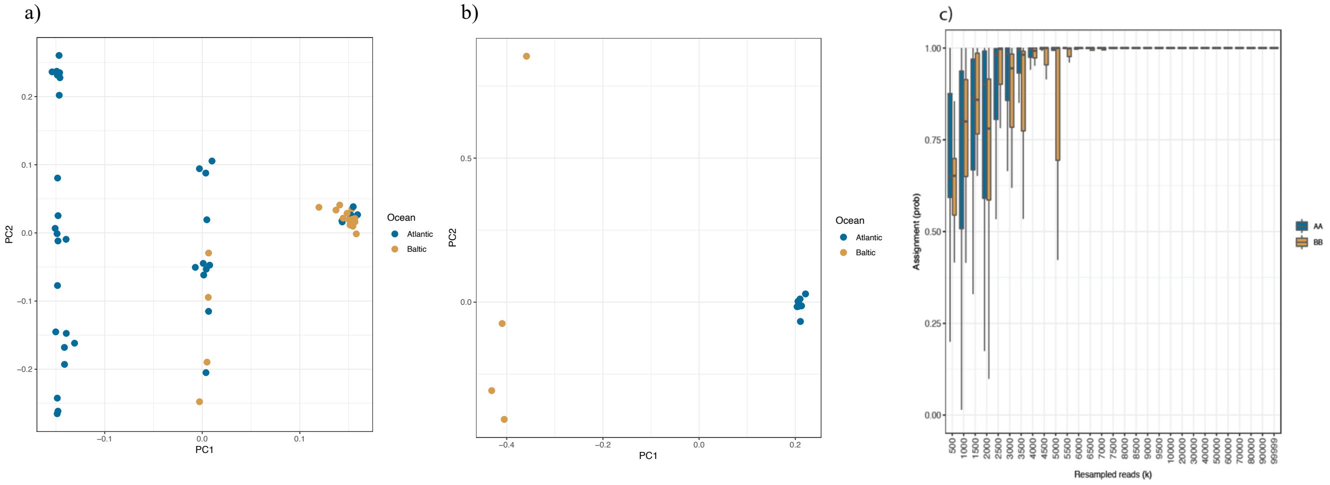

Figure S7 – Chromosome 12 inversion split between Atlantic and Baltic haplotypes. a) Chr12 inversion for all herring individuals in the modern dataset (n=68, Supplementary Dataset S2). b) Chr12 inversion distribution for only autumn spawners only (n=9). This PCA is based up 4503 divergent SNPs. The autumn spawners show a split between the Atlantic and Baltic type inversions, as reported by Han et al.[9] c) The inversion locus within autumn spawners can be scored with as few as 5000 reads. Three of the eight test specimens were autumn spawners, one Baltic type and two Atlantic type (Supplementary Dataset S2). Each individual BAM file from the was randomly down-sampled to between 500 and 100,000 reads for 20 iterations. The y-axis represents the proportion of those files which are known to be type AA or type BB that were assigned correctly using BAMscorer.

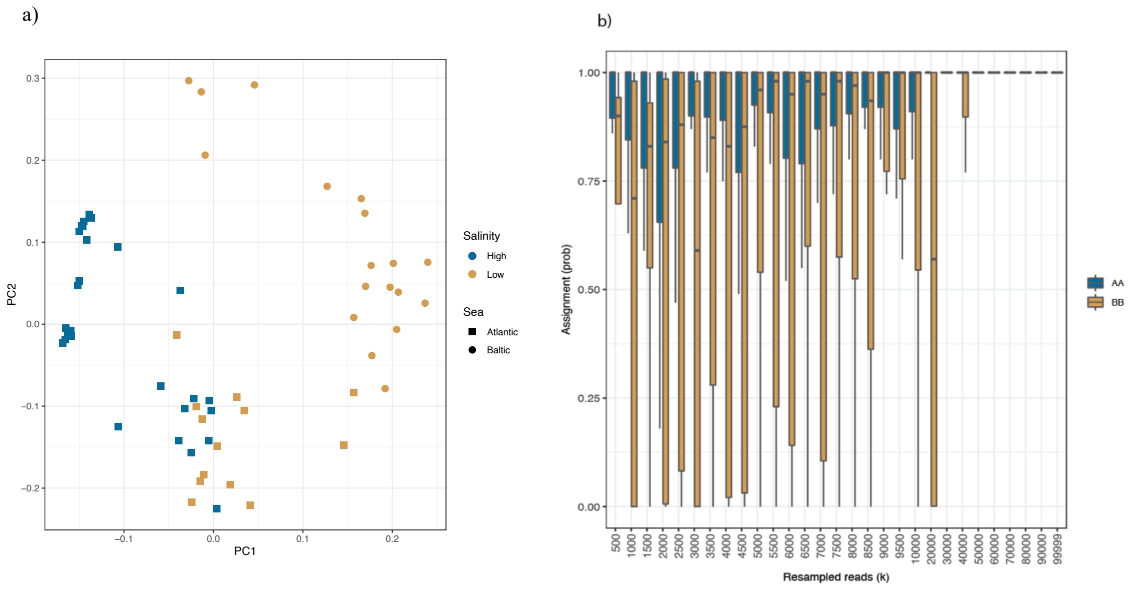

Figure S8 – Salinity adaptation loci BAMscorer database creation and parameter testing. a) PCA generated with smartPCA. Color indicates ocean basin as a proxy for salinity (high being Atlantic, low being Baltic) and shape indicates population of origin. The PCA is based on 2303 divergent SNPs obtained from Han et al.[9] Some Atlantic samples were from Norwegian fjords with low salinity therefore they fell between the two groups. These samples were removed from the dataset for the final scoring. b) Eight individuals with known metadata were removed from the reference dataset for sensitivity analysis (Supplementary Dataset S2). Iterative application of BAMscorer to down-sampled alignment files showing a minimum of 50,000 reads is required for determining salinity adaptation. Each individual BAM file from the eight test specimens training dataset was randomly down-sampled to between 500 and 100,000 reads for 20 iterations. The y-axis represents the proportion of those files which are known to be type AA or type BB that were assigned correctly using BAMscorer.

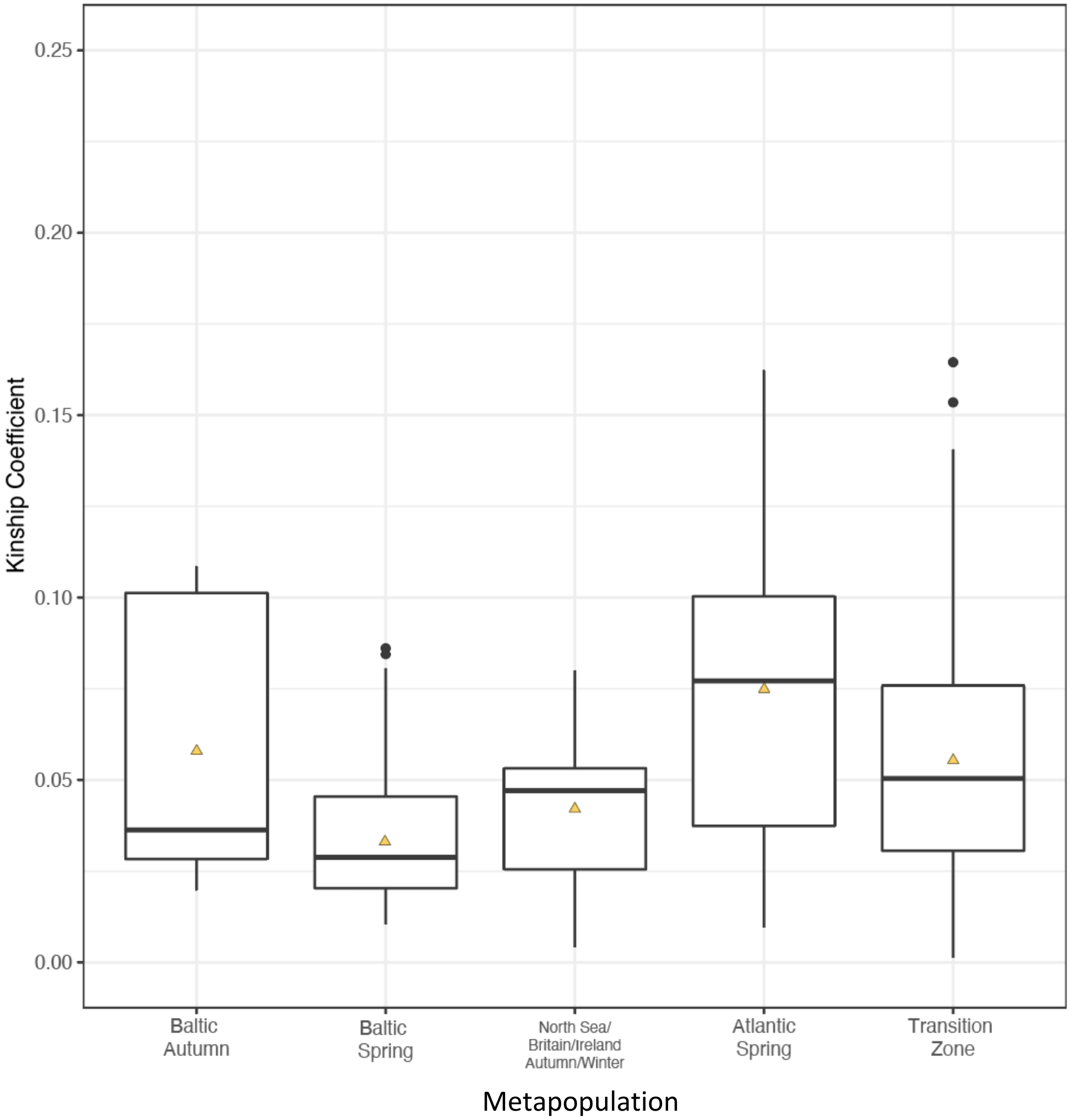

Fig S9 – Individual pairwise relatedness by metapopulation. Each boxplot shows the distribution of kinship coefficients across the metapopulation along the x-axis. Baltic autumn and Baltic spring populations were grouped as two metapopulations to assess the level of substructure in each population; a lower kinship coefficient is indicative of higher substructure. Lines indicate median kinship coefficient and yellow triangles indicate the mean kinship coefficient per metapopulation. Baltic spring spawners show the lowest median and mean kinship coefficient. Baltic autumn show the second-lowest median kinship coefficient, but the mean is dragged higher by the high degree of relatedness in the Fehmarn population.

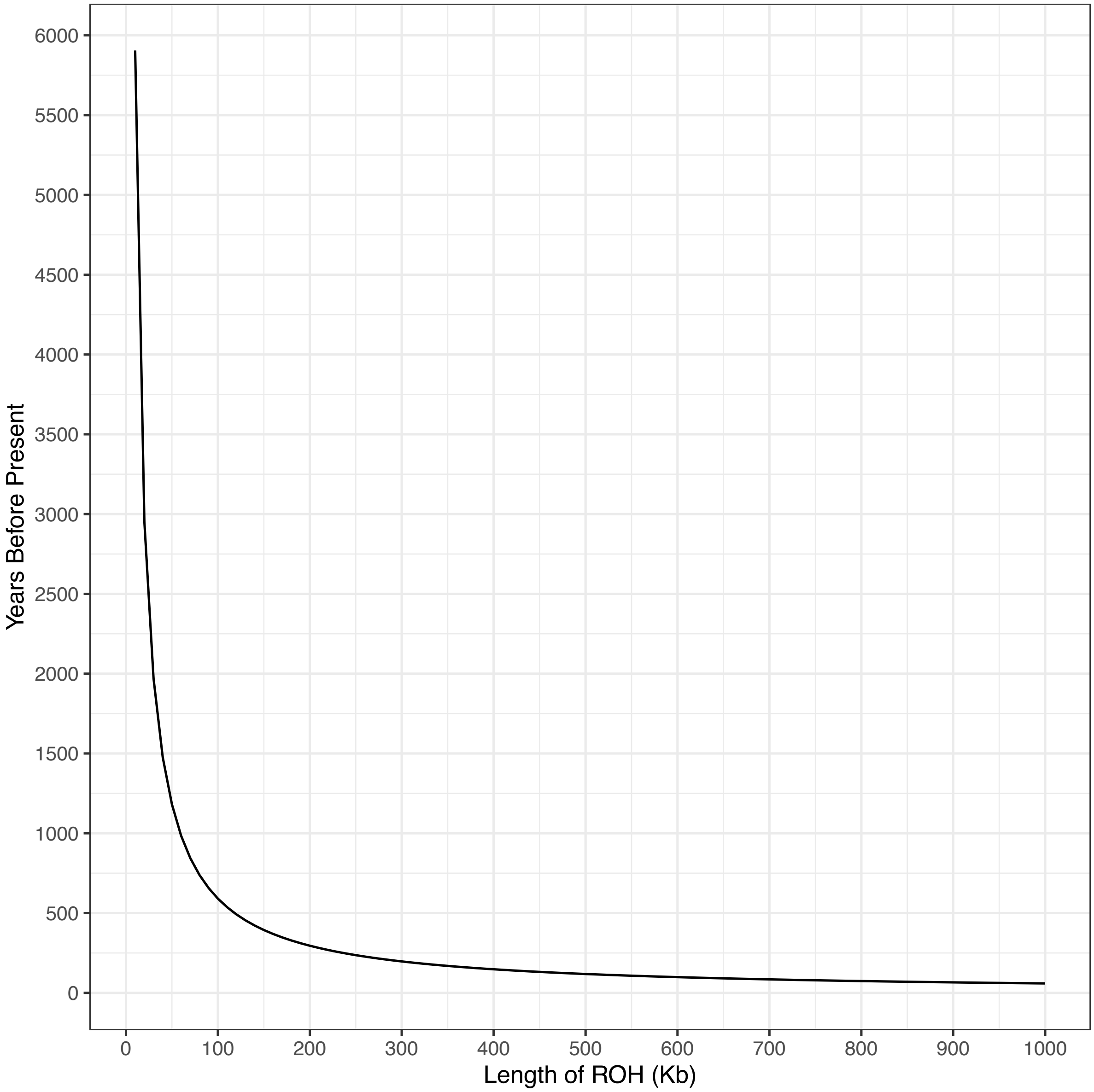

Figure S10 – Time to coalescent event according to length of run of homozygosity. Using the formula 100/2g cM/Mb = L[24] and the known herring recombination rate of 2.54 cM/Mb, we calculated the relationship between length of ROH and timing of coalescent event. The curve indicates the length and its associated coalescent event in years before present.

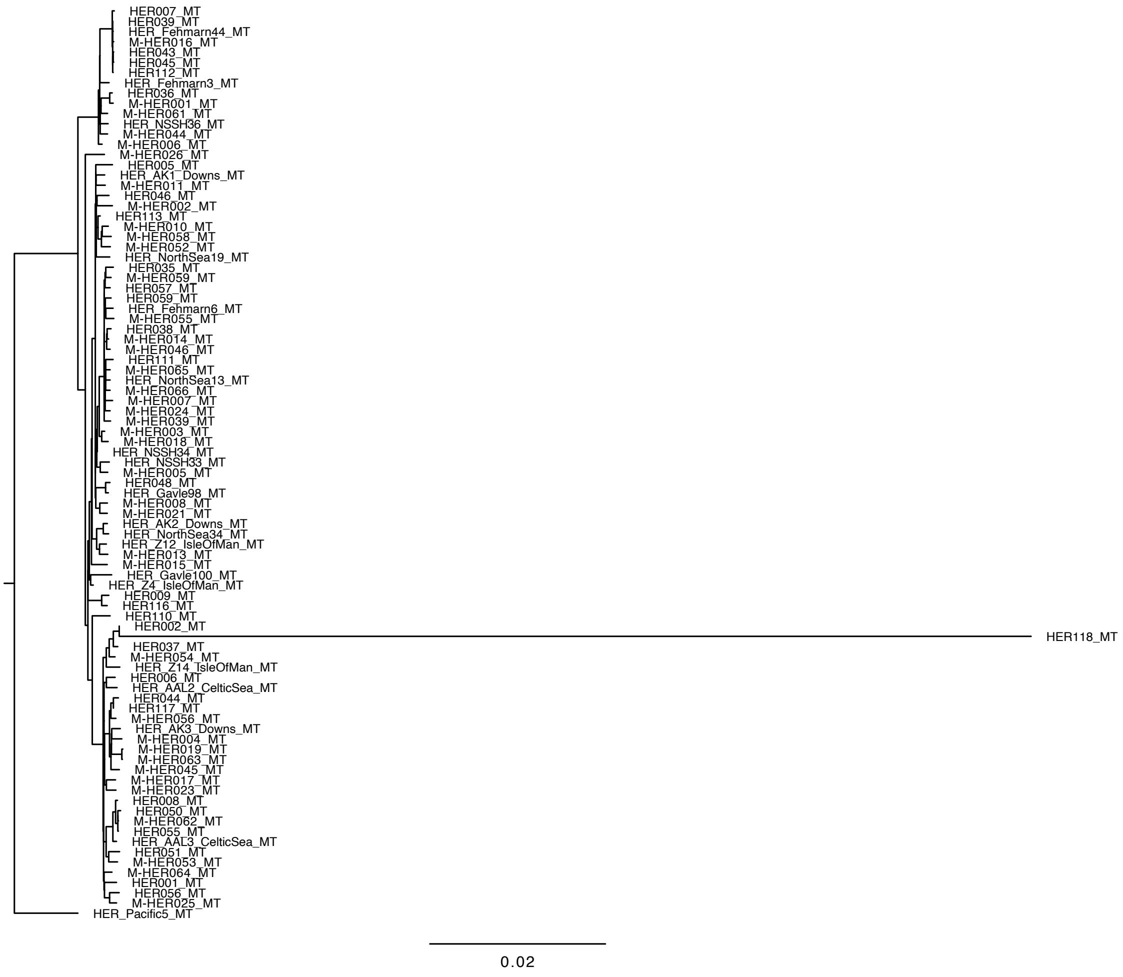

Figure S11 – IQtree phylogeny of the herring mitogenome. All ancient samples clustered with the modern samples and exhibited the structure reported by Teacher et al.[25] that is not associated with geography. HER118 appears quite differentiated from the rest of the samples. BLAST showed that it is indeed a Baltic herring, therefore it was left in the analysis for BAMscorer assignment.

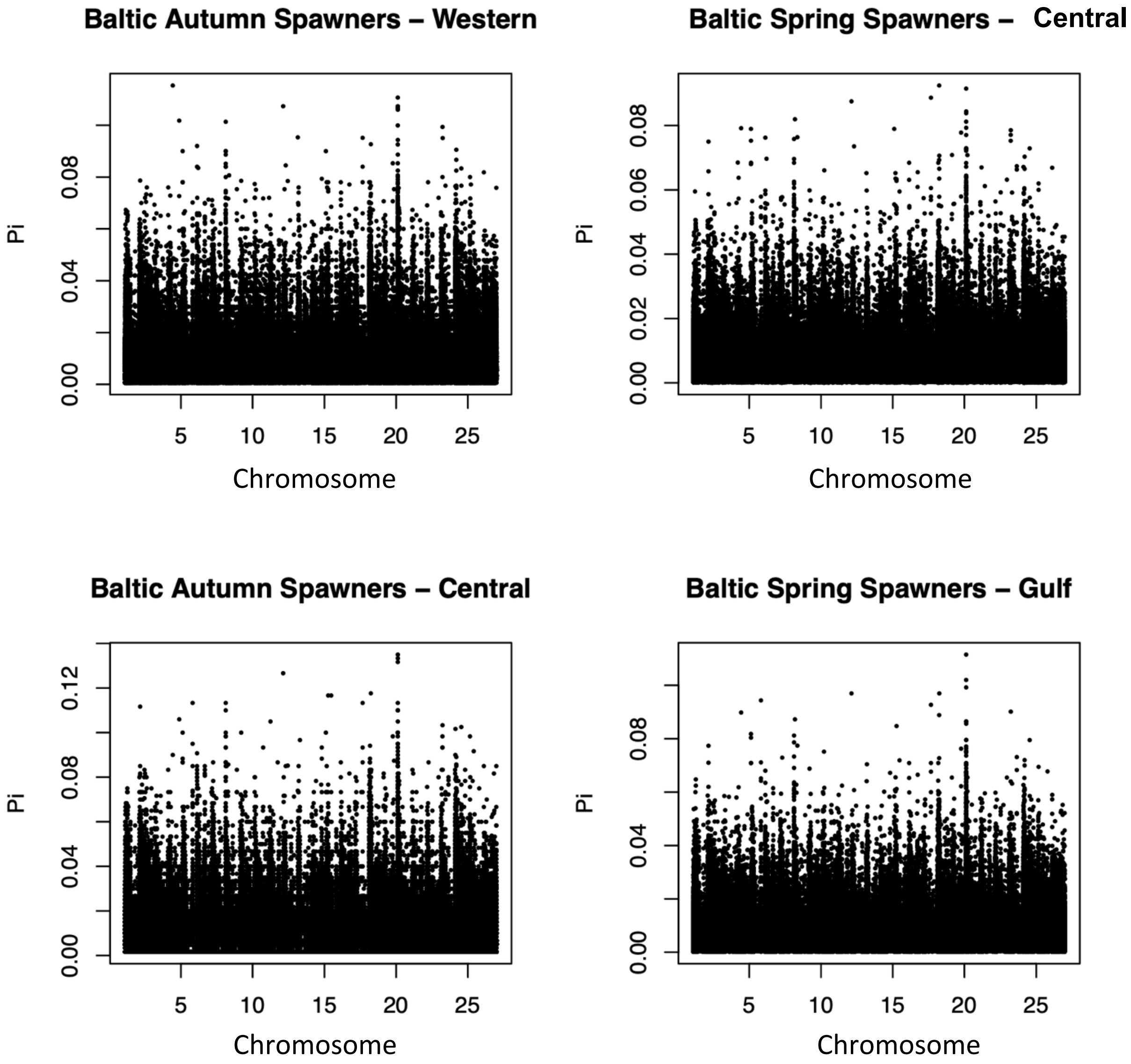

Figure S12 – Estimates of pi for four different Baltic herring populations. The western Baltic autumn spawners had an average pi of 0.006; the central Baltic autumn spawners had an average of 0.008; the central Baltic spring spawners had an average of 0.004; and the gulf spring spawners an average of 0.005.

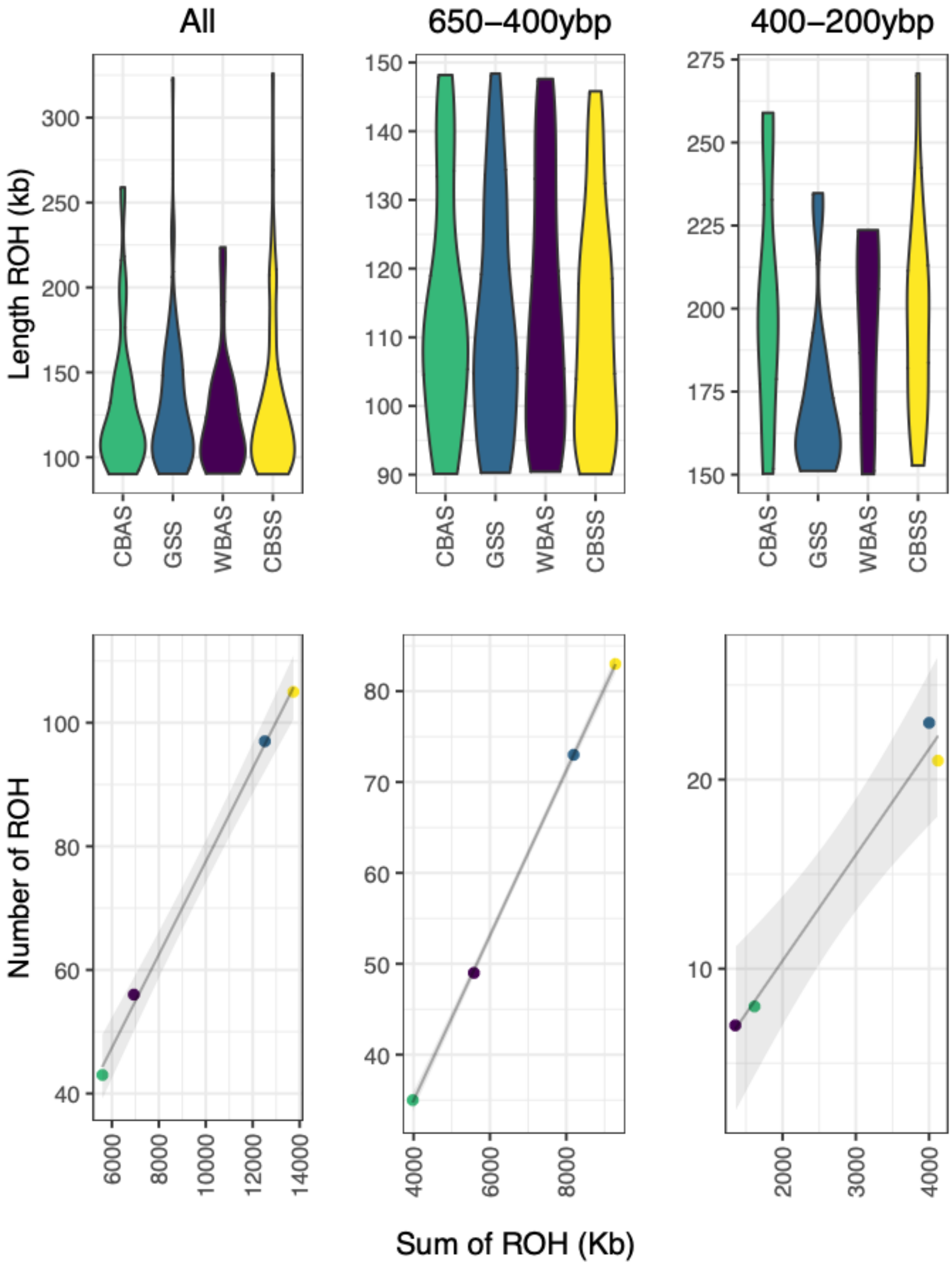

Fig S13 – Runs of homozygosity in four modern Baltic herring stocks. The top panel shows the distribution of ROH across the genome for the entire genome and two bins associated with coalescent events 650-400YBP and 400-200YBP. The bottom panel illustrates the summed ROH for each population compared to the total number of ROH. These results indicate a larger effective population size in the past for both autumn spawning herring populations and a smaller effective population size in the past for both spring spawning herring populations.

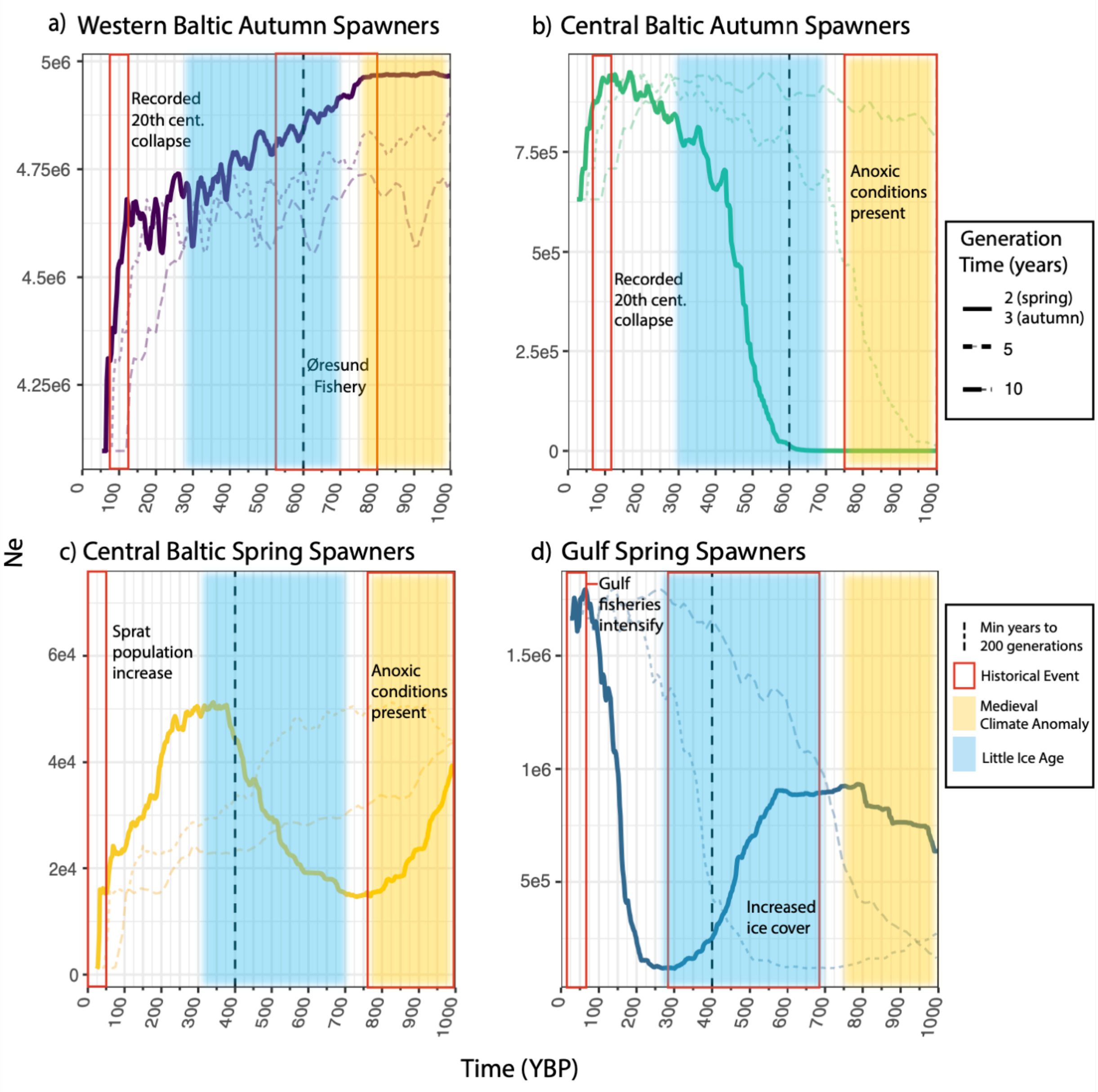
a) Western Baltic Autumn Spawners
Recorded 20th cent. collapse
Øresund Fishery
b) Central Baltic Autumn Spawners
Recorded 20th cent. collapse
Anoxic conditions present
Generation Time (years)
2 (spring)
3 (autumn)
5
10
c) Central Baltic Spring Spawners
Sprat population increase
Anoxic conditions present
d) Gulf Spring Spawners
Gulf fisheries intensify
Increased ice cover
Min years to 200 generations
Historical Event
Medieval Climate Anomaly
Little Ice Age
Ne
Time (YBP)

Fig S14 – gone analysis with generation time uncertainty. Each stock is represented in a separate chart: western Baltic autumn spawners (WBAS); central Baltic autumn spawners (CBAS); central Baltic spring spawners (CBSS); gulf spring spawners (GSS). Colored rectangles indicate key historical events. Yellow rectangles show the approximate duration of the Medieval Climate Anomaly (MCA) and blue rectangles the Little Ice Age (LIA). The dashed vertical lines show the minimum date (YBP) at which 200 generations in the past is reached (calculated using minimum generation times of 3 years for autumn spawners and 2 years for spring spawners), the known accurate window for *gone*. Historical events are denoted by red boxes for each population. As herring reproduce over their lifespan in overlapping generations, additional generation times were used to scale the demography for each population. These are visualized here as dashed lines. a) Demographic trajectory of the western autumn spawners shows a decline starting shortly after the start of the Øresund herring. They show an additional severe decline corresponding to the reported autumn spawning fishery collapse in the Baltic; b) Demographic trajectory of central autumn spawners, which appear limited during the MCA when anoxic conditions are present in the central Baltic. They increase during the LIA and rapidly decline during the period of known autumn spawning population collapse coinciding with the increase of the sprat (*Sprattus sprattus*) population; c) Central spring spawners show an increase around the time of the decline of the western autumn spawners, then a decrease again at the end of the LIA as well as another dramatic decrease around the time of the autumn spawners' collapse ~100YBP; d) Gulf spring spawners decrease during the LIA and then increase dramatically at the end of the LIA, starting to decline only in very recent generations when fisheries in the gulfs intensify. This panel illustrates the accuracy of using generation times between 2-5 years for Baltic herring. GSS were not intensively fished until very recently, therefore their demographic trajectory should reflect changes in environmental factors such as sea surface temperature and presence of ice cover. Generation times 2 and 5 reflect best the expected changes in population size given known responses to such environmental changes in Baltic herring.

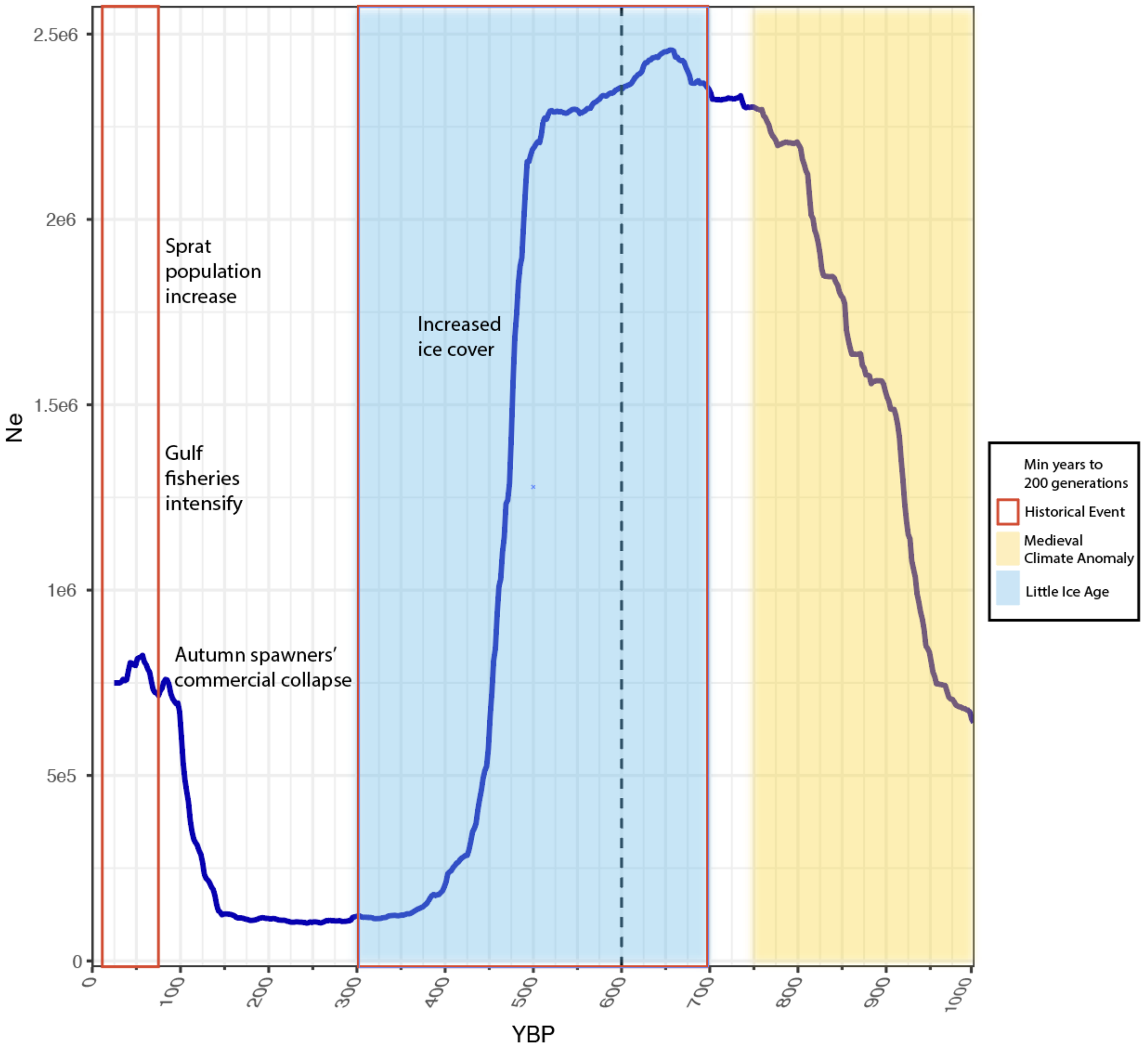

Fig S15 – gone analysis for all Baltic spring spawners. Here, Baltic spring spawners were grouped as a single metapopulation (n=10) and *gone* was run with the same parameters as before. This figure shows the projected demographic trajectory when both the GSS and CBSS are treated as a single population. Historical events are again marked, illustrating the relationship between fishing pressure, changing climate, and demographic trends in Baltic herring.

SI References

1. Damgaard, P. B. *et al.* Improving access to endogenous DNA in ancient bones and teeth. *Sci. Rep.* **5**, 11184 (2015).
2. Meyer, M. & Kircher, M. Illumina Sequencing Library Preparation for Highly Multiplexed Target Capture and Sequencing. *Cold Spring Harb. Protoc.* **2010**, pdb.prot5448 (2010).
3. Kapp, J. D., Green, R. E. & Shapiro, B. A fast and efficient single-stranded genomic library preparation method optimized for ancient DNA. *J. Hered.* doi:10.1093/jhered/esab012.
4. Cooper, A. & Poinar, H. N. Ancient DNA: Do It Right or Not at All. *Science* **289**, 1139–1139 (2000).
5. Gilbert, M. T. P., Bandelt, H.-J., Hofreiter, M. & Barnes, I. Assessing ancient DNA studies. *Trends Ecol. Evol.* **20**, 541–544 (2005).
6. Llamas, B. *et al.* From the field to the laboratory: Controlling DNA contamination in human ancient DNA research in the high-throughput sequencing era. *STAR Sci. Technol. Archaeol. Res.* **3**, 1–14 (2017).
7. Danecek, P. *et al.* Twelve years of SAMtools and BCFtools. *GigaScience* **10**, (2021).
8. Danecek, P. *et al.* The variant call format and VCFtools. *Bioinformatics* **27**, 2156–2158 (2011).
9. Han, F. *et al.* Ecological adaptation in Atlantic herring is associated with large shifts in allele frequencies at hundreds of loci. *eLife* **9**, e61076 (2020).
10. Ferrari, G. *et al.* The preservation of ancient DNA in archaeological fish bone. *J. Archaeol. Sci.* **126**, 105317 (2021).
11. Manichaikul, A. *et al.* Robust relationship inference in genome-wide association studies. *Bioinformatics* **26**, 2867–2873 (2010).
12. Price, A. L. *et al.* Principal components analysis corrects for stratification in genome-wide association studies. *Nat. Genet.* **38**, 904–909 (2006).
13. Patterson, N., Price, A. L. & Reich, D. Population Structure and Eigenanalysis. *PLoS Genet.* **2**, (2006).
14. Wahlund, S. Zusammensetzung Von Populationen Und Korrelationserscheinungen Vom Standpunkt Der Vererbungslehre Aus Betrachtet. *Hereditas* **11**, 65–106 (1928).
15. Inbreeding Coefficient. *GATK* https://gatk.broadinstitute.org/hc/en-us/articles/360035531992-Inbreeding-Coefficient.
16. Miller, J. M. *et al.* Estimating genome-wide heterozygosity: effects of demographic history and marker type. *Heredity* **112**, 240–247 (2014).
17. Lamichhaney, S. *et al.* Population-scale sequencing reveals genetic differentiation due to local adaptation in Atlantic herring. *Proc. Natl. Acad. Sci.* **109**, 19345–19350 (2012).
18. Lamichhaney, S. *et al.* Parallel adaptive evolution of geographically distant herring populations on both sides of the North Atlantic Ocean. *Proc. Natl. Acad. Sci.* **114**, E3452–E3461 (2017).
19. Second-generation PLINK: rising to the challenge of larger and richer datasets | GigaScience | Oxford Academic. https://academic.oup.com/gigascience/article/4/1/s13742-015-0047-8/2707533.

20. Kardos, M., Qvarnström, A. & Ellegren, H. Inferring Individual Inbreeding and Demographic History from Segments of Identity by Descent in Ficedula Flycatcher Genome Sequences. *Genetics* **205**, 1319–1334 (2017).
21. Ceballos, F. C., Joshi, P. K., Clark, D. W., Ramsay, M. & Wilson, J. F. Runs of homozygosity: windows into population history and trait architecture. *Nat. Rev. Genet.* **19**, 220–234 (2018).
22. Foote, A. D. *et al.* Runs of homozygosity in killer whale genomes provide a global record of demographic histories. *Mol. Ecol.* **30**, 6162–6177 (2021).
23. Jónsson, H., Ginolhac, A., Schubert, M., Johnson, P. L. F. & Orlando, L. mapDamage2.0: fast approximate Bayesian estimates of ancient DNA damage parameters. *Bioinformatics* **29**, 1682–1684 (2013).
24. Kirin, M. *et al.* Genomic Runs of Homozygosity Record Population History and Consanguinity. *PLOS ONE* **5**, e13996 (2010).
25. Teacher, A. G., André, C., Jonsson, P. R. & Merilä, J. Oceanographic connectivity and environmental correlates of genetic structuring in Atlantic herring in the Baltic Sea. *Evol. Appl.* **6**, 549–567 (2013).
